# Cell type-specific immune regulation under symbiosis in a facultatively symbiotic coral

**DOI:** 10.1093/ismejo/wraf132

**Published:** 2025-06-26

**Authors:** Maria Valadez-Ingersoll, Hanny E Rivera, Jeric Da-Anoy, Matthew R Kanke, Kelly Gomez-Campo, M Isabel Martinez Rugerio, Sebastian Metz, Michael Sweet, Julian Kwan, Ryan Hekman, Andrew Emili, Thomas D Gilmore, Sarah W Davies

**Affiliations:** Department of Biology, Boston University, Boston, MA 02215, United States; Department of Biology, Boston University, Boston, MA 02215, United States; Ginkgo Bioworks, Boston, MA 02210, United States; Department of Biology, Boston University, Boston, MA 02215, United States; Amgen, Research Biomics, South San Francisco, CA 94080, United States; Department of Biology, State College, Pennsylvania State University, PA 16801, United States; Helmholtz Institute for Functional Marine Biodiversity (HIFMB), 26129, Oldenburg, Germany; Department of Biology, State College, Pennsylvania State University, PA 16801, United States; Helmholtz Institute for Functional Marine Biodiversity (HIFMB), 26129, Oldenburg, Germany; Aquatic Research Facility, Nature-based Solutions Research Centre, University of Derby, Derby, United Kingdom; Aquatic Research Facility, Nature-based Solutions Research Centre, University of Derby, Derby, United Kingdom; Department of Biochemistry, Boston University Chobanian & Avedisian School of Medicine, Boston, MA 02118, United States; Department of Biochemistry, Boston University Chobanian & Avedisian School of Medicine, Boston, MA 02118, United States; Department of Biology, Boston University, Boston, MA 02215, United States; Department of Biochemistry, Boston University Chobanian & Avedisian School of Medicine, Boston, MA 02118, United States; Division of Oncological Sciences, Knight Cancer Institute Oregon Health & Science University, Portland, OR 97201, United States; Department of Biology, Boston University, Boston, MA 02215, United States; Department of Biology, Boston University, Boston, MA 02215, United States

**Keywords:** coral, symbiosis, immunity, single-cell RNA-sequencing, proteomics

## Abstract

Many cnidarians host single-celled algae within gastrodermal cells, yielding a mutually beneficial exchange of nutrients between host and symbiont, and dysbiosis can lead to host mortality. Previous research has uncovered symbiosis tradeoffs, including suppression of immune pathways in hosts, and correlations between symbiotic state and pathogen susceptibility. Here, we used a multiomic approach to characterize symbiotic states of the facultatively symbiotic coral *Oculina arbuscula* by generating genotype-controlled fragments of symbiotic and aposymbiotic tissue. 16S rRNA gene sequencing showed no difference in bacterial communities between symbiotic states. Whole-organism proteomics revealed differential abundance of proteins related to immunity, confirming immune suppression during symbiosis. Single-cell RNAseq identified diverse cell clusters within seven cell types across symbiotic states. Specifically, the gastrodermal cell clusters containing algal-hosting cells from symbiotic tissue had higher expression of nitrogen cycling and lipid metabolism genes than aposymbiotic gastrodermal cells. Furthermore, differential enrichment of immune system gene pathways and lower expression of genes involved in immune regulation were observed in these gastrodermal cells from symbiotic tissue. However, there were no differences in gene expression in the immune cell cluster between symbiotic states. We conclude that there is evidence for compartmentalization of immune system regulation in specific gastrodermal cells in symbiosis. This compartmentalization may limit symbiosis tradeoffs by dampening immunity in algal-hosting cells while simultaneously maintaining general organismal immunity.

## Introduction

Symbioses exist across the Tree of Life along a spectrum from parasitism—in which one symbiotic partner benefits at the cost of the other partner—to mutualism—in which both partners benefit from the relationship [1]. Endosymbioses, symbiotic relationships where one organism resides within another, pose immunity challenges to hosts as they must differentiate between self, mutualistic symbionts, and pathogens [2, 3]. In mutualisms, the host immune system is often down-regulated in order to allow for the existence of the endosymbiont [4], whereas in parasitisms the endosymbiont can evade detection by the host’s immune system [5]. Additionally, in many mutualisms, the hosts must tightly regulate the density of their symbionts in order to prevent overcrowding, which can lead to a shift from mutualism to parasitism [6].

Reef-building corals participate in a mutually beneficial symbiosis with algae (family Symbiodiniaceae) that reside inside their gastrodermal cells, wherein the algae carry out photosynthesis and translocate fixed carbon sugars to the host [7, 8]. In return, the host provides CO_2_, as well as nitrogen and phosphorus species to the symbiont [9, 10]. Translocation of nitrogen from the host to the symbiont has been proposed as a mechanism to promote nutrient exchange while controlling symbiont proliferation via nitrogen limitation to prevent overcrowding [11]. If symbiont densities increase, the greater nutrient requirements of the algae lead to resource competition with the host, thereby destabilizing the partnership [12, 13]. In support of this model, whole-organism RNA-sequencing has revealed differences in the expression of genes involved in sugar transport and nitrogen cycling across cnidarians in and out of symbiosis [14]. Furthermore, enrichment of nitrogen deprivation genes has been observed in the symbionts of the sea anemone *Exaiptasia pallida* when symbiont cell densities are high [11]. These whole organism studies reflect the importance of carbon and nitrogen transport between symbiotic partners in the maintenance of this symbiosis.

In stony corals, environmental change can lead to loss of the algal symbiont in a process termed coral bleaching [15]. If prolonged, this dysbiosis can lead to host mortality. Because this symbiotic relationship in corals is often obligate, it is challenging to disentangle the effects of dysbiosis from nutritional losses [16]. Facultatively symbiotic cnidarian models (which are viable and naturally occur in both symbiotic and aposymbiotic states) are becoming increasingly useful for understanding mechanisms that promote symbiosis establishment, maintenance, and loss, from both nutritional and immune system perspectives [14, 16–18]. In contrast to obligate symbioses, facultatively symbiotic corals can exist in an aposymbiotic state (with few or no algal symbionts) and can buffer nutrient loss through heterotrophy. This facultative nature allows for studies of the influence of symbiosis on host molecular function in the absence of the starvation stress experienced by bleached obligate corals [19]. The aposymbiotic state can be induced in facultatively symbiotic cnidarians in controlled laboratory conditions through chemical (with menthol) and heat treatments, additionally allowing for the ability to control for genetic background [16]. The sea anemone *E. pallida* is a facultatively symbiotic cnidarian that has served as a useful model for understanding how the host immune system is modulated in the presence of the algal symbiont [20, 21]. Additionally, facultatively symbiotic stony corals, such as *O. arbuscula* [14] and *Astrangia poculata* [22], are emerging models for studying these processes in calcifying cnidarians.

Research using these facultatively symbiotic models has identified pathways within the host immune system as key for the cnidarian-algal symbiosis. For example, activation of the transcription factor NF-κB pathway, which has been implicated in immunity across metazoans, has been shown to be downregulated in symbiotic compared to aposymbiotic hosts [14, 20]. Additional evidence suggests that the attenuation of immune pathways associated with symbiosis corresponds to reduced organismal immunity [23]. However, this dampening of immunity is not always correlated with increased susceptibility to pathogen challenge [24, 25]. For example, recent work has shown that the NF-κB pathway can be independently modulated by cnidarians capable of symbiosis under energetic limitation [26]. Additionally, many antioxidant and oxidative response pathways are constitutively higher in symbiotic compared to aposymbiotic cnidarians [27]. Taken together, a nuanced picture of the symbiotic cnidarian immune system is emerging, but questions still remain, especially regarding how the immune system challenge of endosymbiosis is mitigated without compromising whole-organism immunity.

Herein, we have used a multiomic approach to investigate symbiosis-immunity tradeoffs in the facultatively symbiotic coral *O. arbuscula*. First, a gold-standard genome of *O. arbuscula* was assembled. Then, whole-organism proteomics was used to confirm broad immune dampening in symbiosis. Next, single-cell RNA-seq of genotype-controlled fragments profiled *O. arbuscula* cellular diversity under different symbiotic states. We provide evidence for how tradeoffs between endosymbiosis and immunity manifest by gene expression rewiring across different cell states. Gene expression differences in immune pathways occur in gastrodermal cells capable of hosting symbiotic algae. These results suggest that cell type-specific dampening of immunity explains how some symbiotic organisms balance the immune tradeoffs of symbiosis with organismal immunity.

## Materials and methods

Detailed information on sample preparations, bioinformatic analyses, and R packages can be found in the Supplemental Materials.

### Coral husbandry and manipulation of symbiotic state

Symbiotic (in symbiosis with *Breviolum psygmophilum*) [28] colonies from seven genetic backgrounds (genets A-G) of *Oculina arbuscula* were collected at Radio Island Jetty, North Carolina (34° 42.520’ N, 76° 40.796’ W) in May 2018 under North Carolina Division of Marine Fisheries Permit #1627488. These colonies have been maintained at Boston University (BU) in common garden aquaria since May 2018. Aposymbiotic branches were generated via menthol bleaching. Aposymbiotic status was confirmed by a lack of symbiont autofluorescence under fluorescence microscopy (Leica M165 FC), and aposymbiotic branches were transferred back to common garden aquaria and maintained for at least two months of recovery prior to physiological and multiomic profiling.

### Tissue removal for symbiont cell quantification

To measure symbiont cell densities in symbiotic and aposymbiotic *O. arbuscula*, small branches were fragmented from symbiotic (N = 10) and aposymbiotic (N = 10) samples from five genets. Tissue was removed via airbrushing, homogenized, and symbiont counts were normalized to skeletal surface area using an Einscan-SE scanner. Statistical differences between symbiotic and aposymbiotic fragments were calculated using the Kruskal-Wallis rank-sum test, as assumptions of normality were not met.

### Coral spectroscopic determinations

Light absorption capacities of symbiotic and aposymbiotic *O. arbuscula* were compared using reflectance and absorptance [29–31]. Coral reflectance (R), i.e., the fraction of light reflected, was measured between 400–750 nm in intact fragments using a miniature spectroradiometer (Flame-T-UV–Vis, Ocean Optics Inc.). Reflectance was expressed as the ratio of the measurement from the tissue surface relative to the reflectance of a bleached *O. arbuscula* skeleton. Coral absorptance (A), which describes the fraction of incident light absorbed by coral tissue, was calculated from the reflectance spectra as A = 1 – R [29, 31]. The absorptance peak of chlorophyll a (Chl a) at 675 nm was calculated as A_675_ = 1 – R_675_, assuming that transmission through the skeleton is negligible.

### Bacterial community profiling

To identify bacterial communities associated with symbiotic and aposymbiotic fragments, one symbiotic and one aposymbiotic fragment from five genotypes were flash frozen, and a subsample was preserved in ethanol for 16S rRNA gene sequencing (N = 10). Libraries were generated using a series of PCR amplifications for the V4/V5 region of the bacterial 16S rRNA gene [32, 33]. Five negative controls were prepared and used to remove contamination. Samples were sequenced on a Miseq System (Illumina) at Tufts University Core Facility (paired-end 250 bp).

16S rRNA gene read processing inferred 661 amplicon sequence variants (ASVs) with assigned taxonomy across samples. Bacterial communities across symbiotic states were compared via alpha diversity (Shannon index, Simpson’s index, ASV richness, and evenness) and beta diversity (PCoA with Bray-Curtis dissimilarities). Alpha diversity metrics were compared using linear mixed effects models with symbiotic state as the predictor and a random effect of genotype. The effect of symbiotic state on beta diversity was assessed using the function betadisper (vegan package; v2.6–4 [34]). DESeq2 v1.40.2 [35] explored differentially abundant ASVs to ensure that subtle differences in specific taxa were not overlooked.

### *O. arbuscula* genome assembly and annotation

A chromosome-level genome assembly of *O. arbuscula* was recently released and is available from NCBI under the accession number GCA_964656845.1. This genome was generated by the Aquatic Symbiosis Genomics Project (https://www.aquaticsymbiosisgenomics.org) [36] using ~25× coverage PacBio HiFi reads and Arima Genomics Hi-C data. The initial assembly was produced with Hifiasm [37] in Hi-C integrated assembly mode. Scaffolding based on Hi-C contact maps was performed using YaHS [38]. The mitochondrial genome was assembled with MitoHiFi v2.2 [39], and each haplotype assembly was manually curated using TreeVal [40]. Chromosome-scale scaffolds confirmed by Hi-C contact maps were named according to their size.

For structural gene annotation, the genome was first soft-masked using RepeatModeler2 v2.0.5 [41] with the -LTRStruct module enabled. Transcriptomic data were retrieved from GenBank using VARUS [42] and aligned to the genome with HISAT2 v2.2.1 [43]. The soft-masked genome, aligned RNA-seq reads, and a Scleractinia protein dataset containing over 1.2 million sequences were used for gene prediction with BRAKER3 [44]. The resulting gene models were further refined through a second BRAKER3 run, incorporating a curated set of proteins from published Robust clade coral genomes. The final predicted proteome was functionally annotated using EggNOG-mapper v2.1.12 [45], and the completeness of the annotation was assessed using BUSCO v5.8.2 [46] against the Cnidaria_odb12 reference dataset.

### Proteomic profiling

Mass spectroscopy (MS) was used to identify differentially enriched proteins from total protein isolated from fragments of four symbiotic genets and three aposymbiotic genets. Total protein was isolated as described previously [47] and was stored at −80°C before analysis by MS.

For MS, tryptic peptide mixtures were analyzed by nano-scale high-performance liquid chromatography (Proxeon EASY-Nano system, Thermo Fisher Scientific) coupled with online nanoelectrospray ionization tandem MS (Q-Exactive HF-X mass spectrometer; Thermo Fisher Scientific) [48]. For protein identification and analysis, data files were searched using the workflow of MaxQuant version 2.4 (http://www.maxquant.org/) under standard settings using the *O. arbuscula* genome. A false discovery rate (FDR) threshold of 1% was used to filter candidate peptides and protein identifications. Following data filtration, normalization, and clustering with Omics Notebook [49], 2,543 proteins were retained. Differential abundance analysis of proteomic profiles between aposymbiotic and symbiotic samples was based on a moderated t-test [49] in which proteins were considered differentially abundant if they had a Bonferroni adjusted *P* < 0.1. Raw intensity counts were rlog-normalized in DESeq2 [35] and visualized using a Principal Component Analysis (PCA) using vegan v2.6-4 [34]. Effects of symbiotic state and genet were assessed using a PERMANOVA with the adonis2 function in vegan v2.6-4 [34]. Predicted *O. arbuscula* peptides were searched against the human proteome v.11.5 from the STRING v.11 database [50] with an e-value cut-off of 1 × 10^-5^. Protein-protein interactions of select differentially expressed proteins (FDR < 0.1) were retrieved from the STRING v.11 database [50]. Interaction networks were visualized using Cytoscape v.3.7.2 [51].

### Single-cell RNA sequencing

To create single-cell libraries, live cells from one symbiotic and one aposymbiotic branch of genotype F of *O. arbuscula* were sampled for single-cell isolation and 10X cDNA sequencing [52]. A detailed protocol for cell isolation can be found on protocols.io at DOI: https://doi.org/10.17504/protocols.io.rm7vzkx72vx1/v1 and is also described in the Supplemental Materials. To minimize cell death, cells were not sorted prior to processing. Because we did not perform cell sorting through a method such as Fluorescence-activated Cell Sorting, multiplets may be present in our dataset. Cell isolation samples were analyzed by BU’s Single Cell Sequencing Core Facility. The symbiotic sample had a concentration of 2,625 cells/μl and a viability of 81.6%. The aposymbiotic sample had a concentration of 3,313 cells/μl and a viability of 86.4%. The cell viability values are similar to other single cell preparation methods reported in the literature (e.g., viability threshold of 80%, *Nematostella vectensis* [53, 54]).

Libraries were generated following the 10X Genomics Chromium Single Cell 3’v3 protocol, and quality was assessed via a Bioanalyzer. Samples were pooled in equimolar concentration and sequenced on a NextSeq 2000 (Illumina, P3 100 kit) with a target of 50,000 reads per cell. CellRanger (v7.2.0 [55]) processed reads, which were aligned to concatenated genomes of *O. arbuscula* (host) and the algal symbiont *B. psygmophilum* [14]. Reads aligning confidently to the host and symbiont references were used to generate CellRanger output files. Default CellRanger cell calling algorithms were employed.

Following alignment, host reads from symbiotic and aposymbiotic samples were analyzed using Seurat (v.5.0.2) [56]. First, doublets were detected and removed using scDblFinder (v.1.20.2) for each sample independently [57]. Next, datasets were cleaned (genes expressed in fewer than three cells were discarded, as were cells expressing fewer than 200 genes or greater than 3000 genes). Using only host genes, the datasets were then normalized and scaled, and a PCA reduction was performed. No duplicated barcodes were detected. Mitochondrial reads could not be removed, as the reference genomes and transcriptome do not have annotated mitochondrial genomes. Datasets were integrated using the Canonical Correlation Analysis (CCA) Integration method on the PCA reduction using the host genes (nearest neighbors parameters). Cell clusters were identified using 30 dimensions (resolution: 0.55) and visualized using Uniform Manifold Approximation and Projection (UMAP). Marker genes for each cluster were identified using FindAllMarkers (Wilcoxin Rank Sum Test; log_2_fold change threshold: 0.5) and cell types were informed by gene annotations and marker gene comparisons from other cnidarian single-cell datasets (e.g. *S. pistallata* and *Xenia* sp.) (Supplementary Dataset S1) [58, 59]. Expression patterns of top marker genes were assigned to cell clusters using violin plots, bubble plots, and visualization of gene expression in individual cells within the UMAP. In total, 28 cell clusters across seven cell types were identified in the UMAP. Broad transcriptomic differences between cell clusters were assessed by identifying the top 10 genes enriched in each cluster compared to all the other clusters.

To identify cells containing algal symbionts, we identified cells in which over 50% of the total reads corresponded to *B. psygmophilum* genes, and then visualized these cells on the UMAP. To determine how symbiosis alters enrichment of gene pathways at the whole-organism level, and how this differential enrichment is reflected in specific cell types, we performed Mann-Whitney U Gene Ontology (GO) term enrichment analysis on all genes from the full single-cell dataset [60]. Significantly enriched GO terms within the “Biological Process” (BP) GO division were defined with an FDR of <0.1. Significantly over- and under-represented GO terms relating explicitly to the NF-κB pathway, Toll-Like Receptor signaling, Interleukins, and Transforming Growth Factor β were identified (Table S1). Cells in which over 4.5% of the total reads corresponded to genes annotated with the GO terms of interest were visualized on the UMAP of all cells from symbiotic and aposymbiotic *O. arbuscula* samples.

To compare expression profiles of specific cell states between symbiotic and aposymbiotic samples, each cell cluster was independently reclustered, and the effect of symbiotic state on gene expression in each cell state was analyzed. First, the cell cluster of interest (e.g., Immune Cell, Gastrodermis 1) was subsetted, and new variable features were identified. Data were rescaled, and dimensionality reduction was re-run (first 30 dimensions for all clusters, except for Neuron 9 and Neuron 10, for which 20 dimensions were used). Subclusters were identified with resolutions between 0.25 and 0.5 (Table S2). The distribution of cells across subclusters between symbiotic states was visualized using UMAPs. Differentially expressed genes between symbiotic states in all cell clusters were identified using DESeq2 within the FindMarkers function. Additionally, marker genes for each subcluster were identified. To highlight differences in the gene expression of the Immune Cell states, the top 20 genes enriched in each Immune Cell subcluster were identified and plotted (genes without annotated Pfam domains were removed).

We used Monocle 3 (v.1.3.7) [61] to identify transcriptomic trajectories and outcomes for all gastrodermal/agal-hosting cells from symbiotic and aposymbiotic samples. We plotted the UMAPs of the symbiotic and aposymbiotic subsets of Gastrodermis clusters 1-5 and the Algal-Hosting gastrodermal cells and overlaid trajectory graphs, noting nodes and outcomes.

To compare expression of genes involved in lipid transport/production and nutrient cycling in gastrodermal cells between symbiotic and aposymbiotic states, the expression of these genes in the reclustered Gastrodermis 1 and Gastrodermis 2 cells was analyzed. First, normalized expression of genes involved in nitrogen cycling/symbiont density control (Glutamate Dehydrogenase [*g20747*]) and lipid metabolism (Long-Chain Fatty Acid CoA Ligase [*ACSBG1*] and Acyl-Coenzyme A Thioesterase [*ACOT4.2*]) [14] were compared across all cell clusters, then across subclusters and symbiotic states in Gastrodermis 1 and 2 (functions FeaturePlot and VlnPlt in the Seurat package [56]). FeaturePlot [56] was then used to visualize co-expression of these genes across the subclusters of Gastrodermis 1 and Gastrodermis 2 cells from the symbiotic sample (scaling per-cell mean expression to a maximum value of 10). To determine whether the co-expression of genes observed in the symbiotic gastrodermal cells was observed in other cell types, we also visualized co-expression of *g20747*, *ACSBG1*, and *ACOT4.2* across Epidermis 1 and Neuron 1 clusters from symbiotic tissue (scaling per-cell mean expression to a maximum value of 10).

To identify genes driving separation between symbiotic states within each cell cluster, the distribution of cells from a particular cluster was visualized along the first 10 PCs of the PCA used to define the UMAP for that cluster. An analysis of variance determined whether the cells were separated by symbiotic state (symbiotic vs. aposymbiotic) along each PC. In Gastrodermis 1 and Gastrodermis 2 clusters, the top 20 genes driving the distribution of cells along each significant PC (10 genes driving each PC in the positive direction and 10 genes driving each PC in the negative direction) were selected, and genes without annotated Pfam domains were removed. In Gastrodermis 1, PCs 1–4, 6, 7, and 10 were selected, comprising a total of 89 genes. In Gastrodermis 2, PCs 1–5, 8, 9, and 10 were selected, comprising a total of 90 genes. Log_2_fold change values from DESeq2 and the accompanying adjusted P value (<0.05) for each gene were plotted in a bar graph. Genes annotated with immune-function related GO terms were noted, as were genes annotated with the Clusters of Orthologous Genes (COG) annotation “posttranslational modification, protein turnover, and chaperones.”

To compare *O. arbuscula* cells potentially hosting algal symbionts with algal-hosting cells from an obligate coral species (*Xenia* sp. [59]), we identified orthologous genes between *O. arbuscula* and *Xenia* using the Broccoli algorithm using 14 cnidarian proteomes with default parameters [62]. We re-ran Seurat normalization, PCA, and UMAP clustering on the pre-integrated *Xenia* dataset from non-regeneration samples [59] to identify 16 clusters (resolution of 0.18 and 30 dimension), including the previously characterized algal-hosting cell cluster and the other two gastrodermal cell clusters. We identified differentially expressed genes between the algal-hosting cells and the gastrodermal cells (adjusted *P* < 0.05 from the FindMarkers function, using both gastrodermal cell clusters as the comparison to the algal-hosting cell cluster), and subsetted these genes for those that had orthologs to *O. arbuscula*. Additionally, we identified differentially expressed genes between symbiotic *O. arbuscula* cells from Gastrodermis 1/Gastrodermis 2/Gastrodermis 3/Gastrodermis 4/Algal-Hosting and aposymbiotic *O. arbuscula* cells from Gastrodermis 1/Gastrodermis 2/Gastrodermis 3/Gastrodermis 4/Algal-Hosting (adjusted P value <0.05 from the FindMarkers function). Gastrodermis 5 was not included because this cluster was not on the same transcriptomic trajectory in symbiotic or aposymbiotic *O. arbuscula*. We subsetted these genes to include only *Xenia* orthologs. We compared which genes among these two sets of orthologs (*Xenia*-sym-to-gastro and *Oculina*-sym-to-apo respectively) were differentially expressed using a Venn diagram [63], creating three groups of differentially expressed orthologs (DEOs). We performed Fisher Exact Tests (DEO presence/absence) [60] to identify enrichment differences across these groups of DEOs in the BP GO division.

## Results

**Figure 1:**
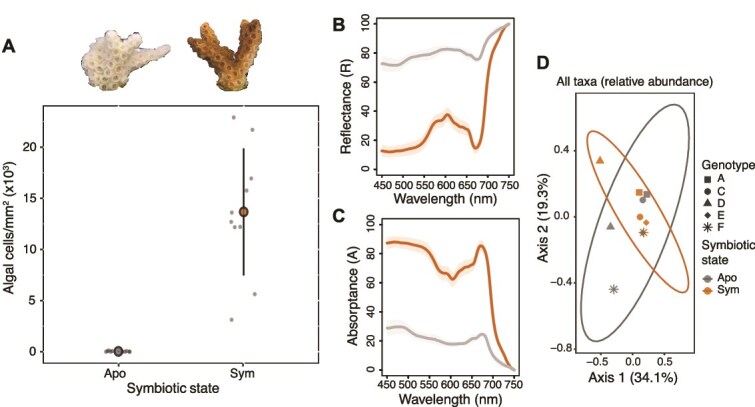
Symbiosis facilitates higher light absorptance but does not change bacterial communities in *O. arbuscula*. (A) Algal cell counts are ~400-fold greater in symbiotic (brown, right) *O. arbuscula* fragments than in aposymbiotic (white/grey, left) fragments (P value <0.001). (B) Average reflectance (R) in the PAR region (400–700 nm) was 22% +/− 4% in symbiotic *O. arbuscula* (brown) and 77% +/− 3% in aposymbiotic *O. arbuscula* (grey) (*P* < 0.01). (C) Average absorptance (A) (A = 1-R) indicating the relative amount of solar energy with potential for use in photosynthesis. Symbiotic *O. arbuscula* (brown) has an A_675_ of 85% (+/− 4%), suggesting a functional symbiosis, and aposymbiotic (grey) has an A_675_ of 24% (+/− 2%) (*P* < 0.001). In (B) and (C), shaded colored areas represent standard deviation. (D) PCoA of bacterial communities from all ASVs in 16S rRNA gene data from symbiotic (brown) and aposymbiotic (grey) *O. arbuscula* genotypes. No differences in bacterial communities were observed between symbiotic states.

For the following experiments, genetically controlled symbiotic and aposymbiotic fragments of *O. arbuscula* were used. Symbiotic fragments had symbiont densities that were over 400-fold greater (13,678 +/− 6,204 cells/cm^2^) than those of aposymbiotic fragments (30 +/− 43 cells/cm^2^) (P value <0.001) (Fig. 1A). These fragments were then used to generate light-scattering profiles, 16S rRNA gene sequencing libraries, tissue proteomics, and single-cell profiles.

### Light absorption is greater in symbiotic than in aposymbiotic *O. arbuscula*

Differences in symbiont cell densities induced significant differences in *in vivo* coral light absorption. Reflectance spectra measurements and absorption properties of intact corals (describing the relative amount of solar energy/incident light that can potentially be used in photosynthesis for organic carbon fixation) showed statistically significant pigment content differences between symbiotic and aposymbiotic *O. arbuscula*. Average reflectance in the PAR region (400–700 nm) of symbiotic fragments was 22% +/− 4%, whereas aposymbiotic coral reflectance was 77% +/− 3% (P value <0.01) (Fig. 1B). In symbiotic fragments, the absorptance value at the peak for Chlorophyll a (A_675_) was 85% +/− 4%, compared to 24% +/− 2% in aposymbiotic samples (*P* < 0.0001) (Fig. 1C).

### Bacterial communities do not differ between symbiotic states

We profiled the bacterial communities of aposymbiotic and symbiotic fragments from five genets (N = 10). A total of 208,330 sequences were acquired with a mean depth of coverage of 20,833 +/− 16,050 per sample (Table S3). Sample rarefication yielded a total of 8,298 reads per sample with a total of 661 ASVs identified across samples. No differences in bacterial communities (beta diversity) were observed between symbiotic and aposymbiotic fragments (Fig. 1D), and both states were dominated by *Proteobacteria* and *Bacteroidota* (Fig. S1). Additionally, no differences in alpha diversity were observed between symbiotic states regardless of the metric tested (Fig. S2a-d). DESeq2 tested for differentially abundant ASVs across symbiotic states, while modeling genetic background, but no ASVs were identified.

### Assembly and annotation of a high-quality *O. arbuscula* genome

The *O. arbuscula* genome comprises ~515 megabase pairs (Mb), organized into 14 chromosomes and a total of 476 scaffolds. The assembly has a GC content of 38.5%, consistent with other scleractinian corals. A total of 35,006 proteins and 30,354 genes were predicted. Genome completeness, as assessed using BUSCO with the Cnidaria_odb12 reference dataset, was 98.5%, with 83.6% of single-copy and 15% of duplicated BUSCOs, indicating a high-quality assembly and annotation. Approximately 80% of the predicted proteome was functionally annotated using EggNOG-mapper.

### *O. arbuscula* proteome is affected by symbiotic state

**Figure 2:**
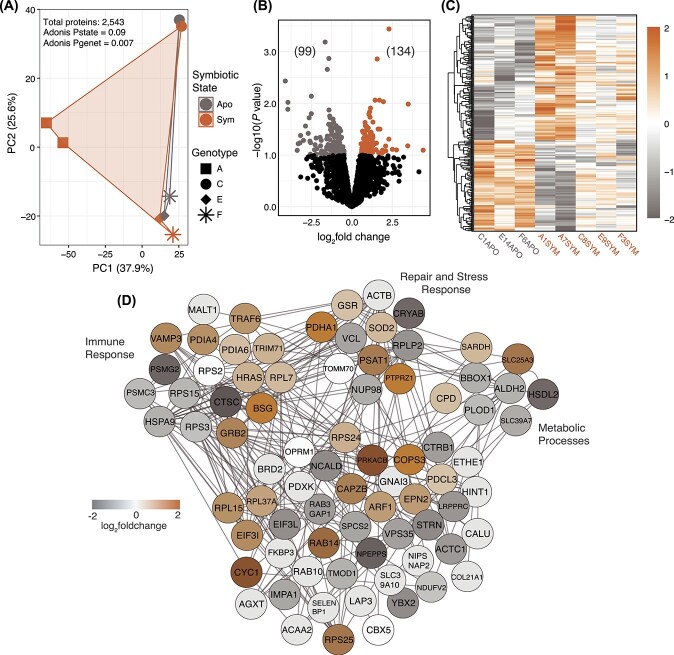
Proteomic profiles of symbiotic and aposymbiotic *O. arbuscula* reveal differential enrichment of proteins by symbiotic state. (A) Principal component analysis of proteomic profiles across four genets, with symbiotic (sym) and aposymbiotic (apo) states distinguished by color, and genets by shape. (B) Volcano plot of differentially enriched proteins (DEPs) identified through pairwise comparison (*P* < 0.1). Upregulated proteins (134) are represented by brown dots, downregulated proteins (99) by grey dots, and black dots indicate non-DEPs in symbiotic relative to aposymbiotic corals (total N = 2,543). (C) Heatmap of all DEPs across symbiotic states of *O. arbuscula*. The color scale represents the log_2_fold change of each protein (row) for each coral sample (column) relative to the protein’s mean expression value across all samples. (D) Interaction network of proteins associated with immune response, repair and stress response, and metabolic processes, derived from human protein-protein interactions in the STRING database. Node colors indicate upregulation (brown) or downregulation (grey) in symbiotic coral samples.

To compare proteomes of symbiotic and aposymbiotic *O. arbuscula*, we performed mass spectrometry on whole-cell lysates from coral fragments. We identified a total of 2,695 proteins in *O. arbuscula*, 2,543 of which were retained following filtering, i.e., ~7.3% of the genome-predicted proteins. A PCA showed that samples clustered by symbiotic state (P_state_ < 0.1) and genet (P_genet_ < 0.05) (Fig. 2A). A total of 233 proteins were differentially enriched across symbiotic states (adjusted P value <0.1): 134 enriched in symbiotic and 99 enriched in aposymbiotic *O. arbuscula* (Figs. 2B-C).

Differentially enriched proteins (DEPs) were assigned functional categories based on a homology search of each DEP sequence in the UniProtKB database (evalue <1 × 10^-5^). DEPs involved in Immune Response, Repair and Stress Response, and Metabolic Processes were identified (Fig. 2D), including multiple heat-shock proteins enriched in aposymbiotic *O. arbuscula* (e.g. HSPA9 and CRYAB) and other immune/stress-related proteins enriched in symbiotic *O. arbuscula* (e.g.TRAF6 and SOD2) (Fig. 2D).

### Identification of cell states shared between symbiotic and aposymbiotic *O. arbuscula*

To profile gene expression in different cell types and states from symbiotic and aposymbiotic *O. arbuscula*, we used the 10X single-cell RNA-sequencing (scRNA-seq) platform. We captured a total of 6,951 cells (3,123 cells from the symbiotic sample; 3,828 cells from the aposymbiotic sample), with an average of 158,688 reads per cell, 1,129 median UMI counts per cell, and 650 median genes per cell. The average number of reads was 540,436,426; the average percentage of valid barcodes was 94.8%; the average percentage of valid UMIs was 99.9%; and the average sequencing saturation was 89.9%. 190 doublets were detected and removed from the symbiotic dataset, and 275 doublets were detected and removed from the aposymbiotic dataset. After additional high and low count cells were removed, the symbiotic dataset had a final cell number of 2,912, and the aposymbiotic dataset had a final cell number of 3,546. Further quality control data for both samples are summarized in Supplementary Dataset S2.

**Figure 3:**
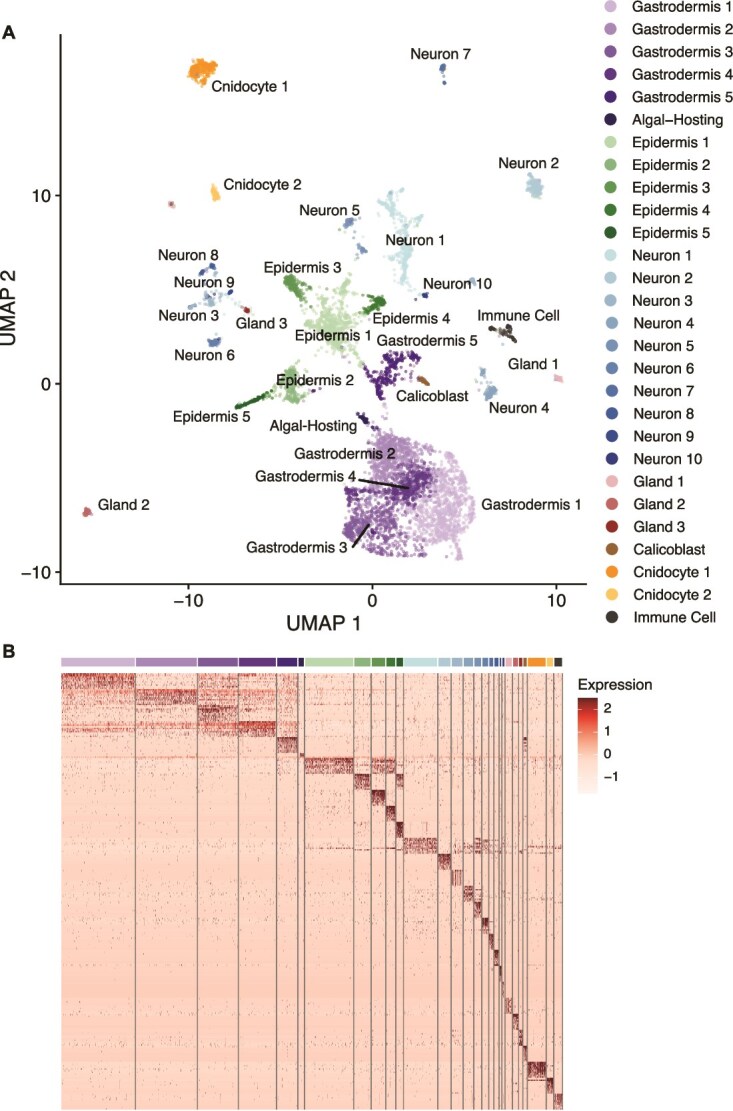
scRNA-seq identifies 28 transcriptomically distinct cell clusters across seven cell types in symbiotic and aposymbiotic *O. arbuscula*. (A) A UMAP projection of the transcriptomes of 6,458 cells from symbiotic (sym) and aposymbiotic (apo) *O. arbuscula* identifies 28 clusters across seven broad cell types. Each cluster is color coded. (B) Each cluster is transcriptomically distinct, with expression similarities present between clusters of the same cell type, as grouped by the 10 genes most highly expressed in each cluster. Expression values are the average log_2_fold changes computed from normalized gene expression data for each cell. Cell clusters are colored as in (A).

Clustering of the integrated 10X gene expression datasets from symbiotic and aposymbiotic samples resulted in the identification of 28 transcriptomically distinct cell clusters across seven broad cell types (Fig. 3A). Cells from symbiotic and aposymbiotic samples were represented in all clusters (Fig. 3A). Additionally, each cluster defined in the UMAP displayed distinct transcriptomic programming as exemplified by expression patterns of the 10 highest enriched genes in each cluster (Fig. 3B).

### Identification of symbiont-hosting cells

We identified 45 cells in which over 50% of the total reads corresponded to genes from B. psygmophilum. 42 of these 45 cells belonged to the same cell cluster from symbiotic samples. Due primarily to the assignment of the symbiont reads to these cells, this cluster was identified as Algal-Hosting gastrodermal cells (Fig. S3).

### Symbiotic gastrodermal cell populations fated for symbiosis function in nutrient exchange

**Figure 4:**
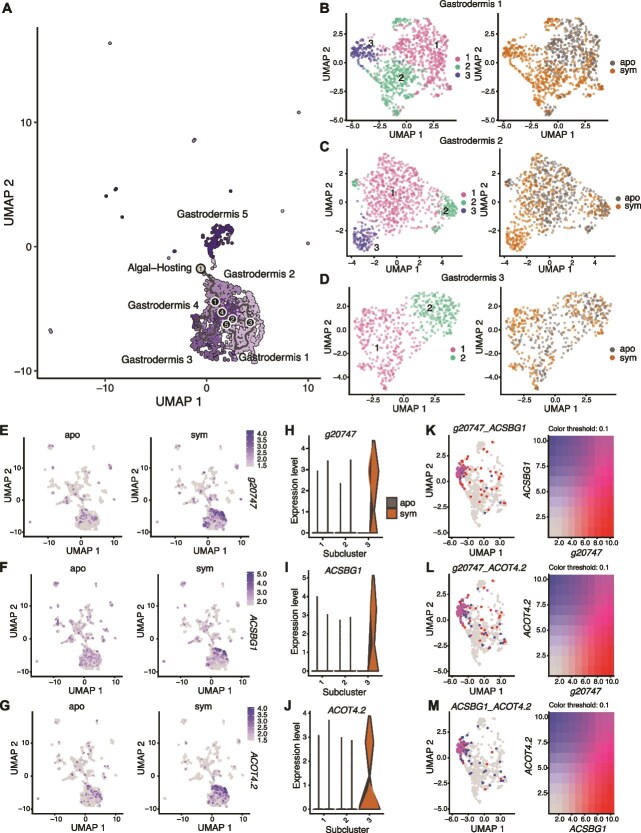
Gastrodermis cells along the algal-hosting trajectory are involved in lipid metabolism and nitrogen cycling. (A) UMAP projection of Gastrodermis 1, 2, 3, 4, and 5 as well as algal-hosting cells from symbiotic *O. arbuscula* overlaid with a graph of cell trajectories. Outcomes (fates) are denoted by grey circles with black numbers. Branch nodes are denoted by black circles with white numbers. (B) UMAP projections of the 1,019 independently reclustered Gastrodermis 1 cells revealed three subclusters. Each subcluster is color-coded in the left panel. Gastrodermis 1 cells separate according to symbiotic state across the UMAP projection (right panel). (C) UMAP projections of the 837 independently reclustered Gastrodermis 2 cells revealed three subclusters, color coded in the left panel. Gastrodermis 2 cells separate according to symbiotic state (right panel). (D) UMAP projections of the 547 independently reclustered Gastrodermis 3 cells revealed two subclusters, color coded in the left panel. Limited separation of Gastrodermis 3 cells by symbiotic state (right panel) was observed. Spatial representation of the gene expression of a putative Glutamate Dehydrogenase (*g20747*, E), Long-Chain Fatty Acid CoA Ligase (*ACSBG1*, F), and Acyl-Coenzyme A Thioesterase (*ACOT4.2*, G) across all cell clusters in both symbiotic states. Coloration represents per-cell-normalized expression of each gene. Expression of *g20747* (H), *ACSBG1* (I), and *ACOT4.2* (J) were compared across subclusters and symbiotic states of Gastrodermis 1. Expression levels of genes in (H–J) are normalized per cell. Co-expression plots of *g20747* and *ACSBG1* (K), *g20747* and *ACOT4.2* (L), and *ACSBG1* and *ACOT4.2* (M) across Gastrodermis 1 cells from symbiotic *O. arbuscula* revealed significant co-expression of these nitrogen cycling and lipid metabolism genes within Subcluster 3, but not in the other subclusters. The coloration of each cell represents the per-cell mean expression value of each gene scaled to a maximum value of 10.

To investigate the transcriptomic fates of the gastrodermal cells, we performed trajectory analysis on the six gastrodermal cell clusters (Gastrodermis 1-5 and Algal-Hosting cells) in the symbiotic and aposymbiotic *O. arbuscula* samples. In the symbiotic sample, we identified one transcriptomic fate terminating in the Algal-Hosting cell cluster (Fig. 4A). Three fates were identified in the aposymbiotic gastrodermal cells, terminating in Gastrodermis 1 (two fates) and 2 (one fate) (Fig. S4).

To probe gene expression differences between gastrodermal cells from symbiotic and aposymbiotic *O. arbuscula*, we subsetted and reclustered cells from each of the six gastrodermal states. Three subclusters were revealed within Gastrodermis 1 (Fig. 4B), three subclusters were revealed within Gastrodermis 2 (Fig. 4C), two were revealed in Gastrodermis 3 (Fig. 4D), two were revealed within Gastrodermis 4 (Fig. S5A), two were revealed in Gastrodermis 5 (Fig. S5B), and two were revealed in the Algal-Hosting cells (Fig. S5C). The presence of these transcriptomically distinct subclusters suggests additional functional cell states. Gastrodermis 1 Subcluster 3 and Gastrodermis 2 Subcluster 3 were composed almost entirely of cells from the symbiotic sample (Fig. 4B and Fig. 4C, respectively). Additionally, Subcluster 1 of the Algal-Hosting cells contained 42 cells with over 50% *B. psygmophilum* reads. We independently reclustered all other cell types and observed varying degrees of separation by symbiotic state, but no other subcluster was completely dominated by cells from either symbiotic state (Fig. S5d-x).

To help identify gastrodermal cells participating in active symbiotic nutrient exchange within our dataset, we selected lipid metabolism and nitrogen cycling genes, which have been previously shown to have higher expression in symbiotic compared to aposymbiotic *O. arbuscula* [14]. DESeq2 identified differences in gene expression in gastrodermal cells between symbiotic and aposymbiotic samples, which showed that Gastrodermis 1 cells from symbiotic *O. arbuscula* have higher expression of genes related to nitrogen cycling (*g20747*) and lipid metabolism (*ACSBG1* and *ACOT4.2*) (adjusted P value <0.05) (Fig. 4E-G). Furthermore, *g20747*, *ACSBG1*, and *ACOT4.2* were identified as marker genes for Subcluster 3 (indicating that these genes were more highly expressed inside than they were outside Subcluster 3 (adjusted P value <0.05) (Fig. 4H-J). Additionally, there was strong co-expression of these symbiosis-marker genes in Subcluster 3, suggesting that this cell state is actively processing and storing lipids while mediating the growth and division of algal symbionts (Fig. 4K-M). There was less co-expression of these genes in Subclusters 1 and 2 of Gastrodermis 1, suggesting that other gastrodermal cell states perform nitrogen and lipid processing independently or through distinct pathways. Similar, though weaker, patterns of co-expression were observed in Gastrodermis 2 (Fig. S6a-c). No or limited co-expression of *g20747*, *ACSBG1*, and *ACOT4.2* was observed in symbiotic Epidermis 1 and Neuron 1 cells (Fig. S6d-i). The presence of symbiotic-only subclusters within Gastrodermis 1 and 2, coupled with the co-expression of highly expressed markers for symbiosis within one Gastrodermis 1 subcluster suggest that certain gastrodermal cells states are actively hosting symbionts. Observed expression of *g20747*, *ACSBG1*, and *ACOT4.2* was low to undetectable in the Algal-Hosting cell cluster, likely due to the low percentage of host reads within these cells.

### No differences in gene expression in the immune cell cluster between symbiotic states

**Figure 5:**
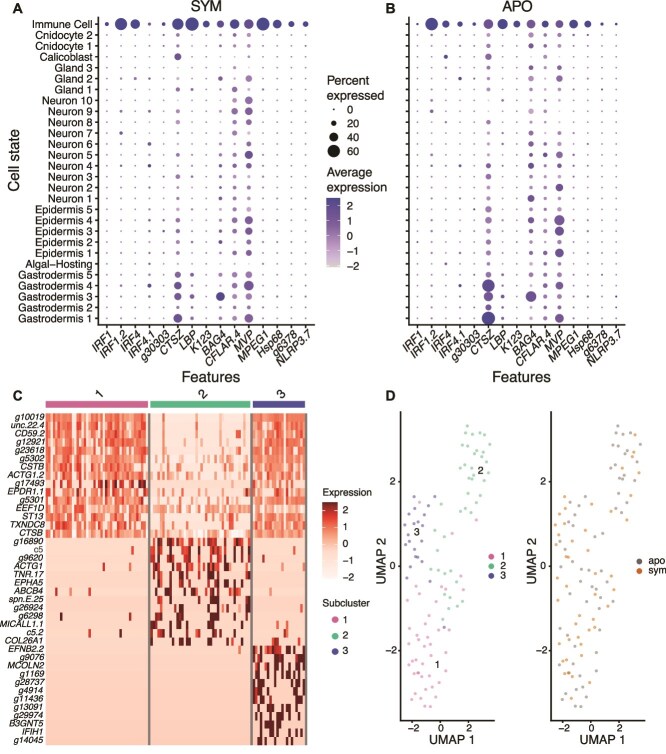
Negligible gene expression differences between cells in the immune cell cluster of symbiotic and aposymbiotic samples. Immune cells from symbiotic (A) and aposymbiotic (B) samples have high expression of previously identified immune cell marker genes. Dot size represents the percentage of cells in each cell state that express each gene, and dot color represents the average (normalized) expression of each gene within each cell state. (C) Three immune cell subclusters have distinct transcriptomic profiles, as represented by the annotated genes within the top 20 most highly enriched genes in each subcluster. Expression values are average log_2_fold changes computed from normalized gene expression data of each cell. Genes with no annotated Pfam domains were removed. (D) UMAP projections of the 107 independently reclustered immune cells revealed three subclusters (left panel) that do not separate according to symbiotic state (right panel).

Whole-organism studies in *O. arbuscula* (this study and [14]) have reported downregulation of genes involved in molecular immunity in symbiotic compared to aposymbiotic coral. To determine whether this difference in gene expression was observed in *O. arbuscula* immune cells, we first identified an Immune Cell cluster based on enriched expression of cell markers for immunity. These immune marker genes included Interferon Regulatory Factors (IRFs), and multiple chaperone proteins that were expressed in cells from both symbiotic and aposymbiotic tissue (Fig. 5A, B). We did not identify cell type- or symbiotic state-specific expression of other commonly studied cnidarian immune genes, including *NFκB*, *cGAS*, and *STING* [20, 64]. After subsetting and independently reclustering these Immune Cells, we identified three cell states with distinct transcriptomic profiles (Fig. 5C). However, Immune Cells did not cluster by symbiotic state after reclustering (Fig. 5D). Furthermore, cells did not separate significantly by symbiotic state along any of the first 10 Principal Components, and no differential gene expression (adjusted P value <0.05) was observed between Immune Cells from symbiotic and aposymbiotic samples. These results indicate that symbiotic state does not affect gene expression in *O. arbuscula* Immune Cells, and that differential expression of immune genes between symbiotic states must reside in non-traditional immune cells.

### Immune response genes are differentially regulated in symbiotic Gastrodermis 1 and 2 cells

**Figure 6:**
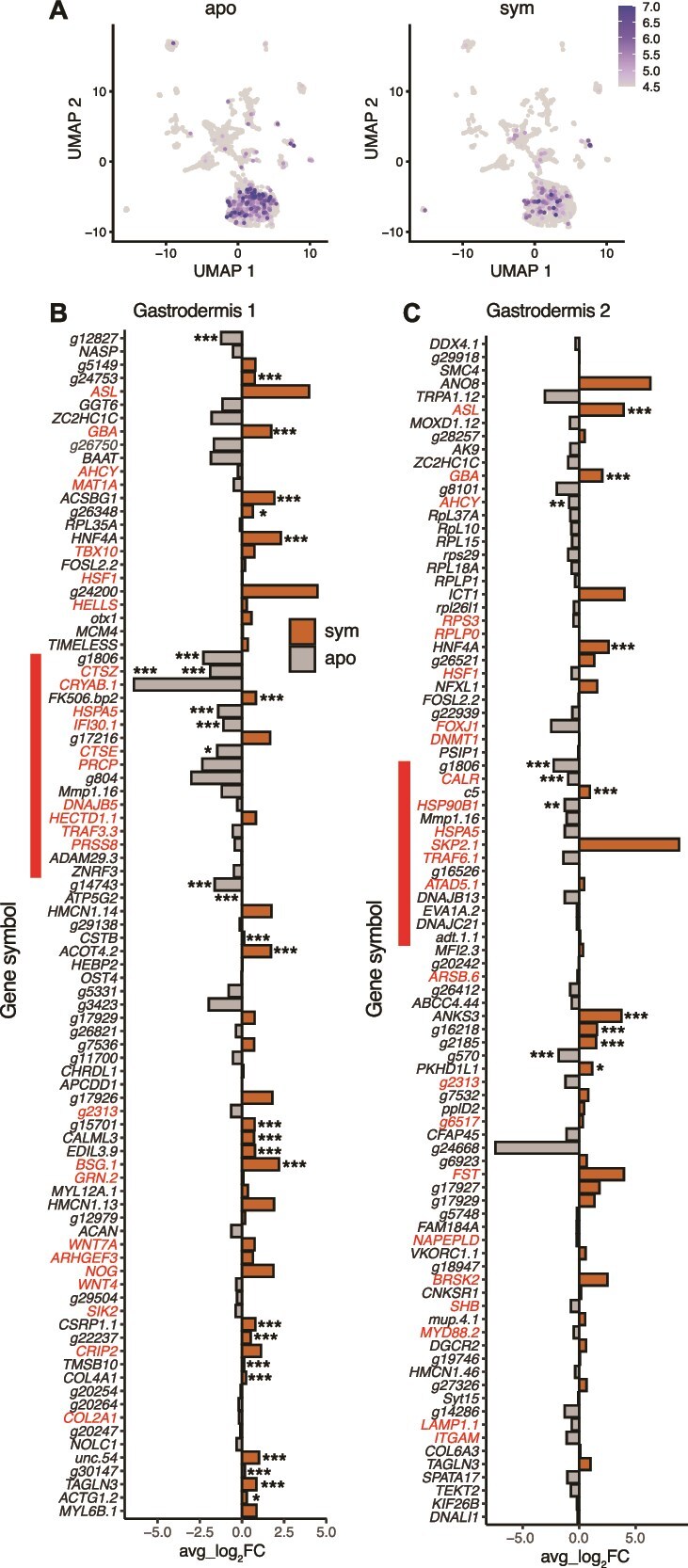
Gastrodermis 1 and 2 cells from symbiotic samples have downregulated immune pathway genes as compared to aposymbiotic samples. (A) Spatial representation showing expression of genes annotated with differentially enriched immunity GO terms across the whole dataset. Coloration represents the percentage of all transcripts in each cell corresponding to the genes of interest (255 genes of interest out of 20,719 total *O. arbuscula* genes with non-zero reads counts). (B) Seven PCs, driven by 89 genes (with annotated Pfam domains), contributed to the separation of symbiotic state in the reclustered UMAP of Gastrodermis 1 (see Fig. 4B). 29 of the 89 were differentially expressed. 27 genes are annotated with GO terms relating to immunity (red text). (C) Eight PCs, driven by 90 annotated genes, contributed to the separation of symbiotic state in the reclustered UMAP of Gastrodermis 2 (see Fig. 4C). 12 of the 90 genes were differentially expressed. 25 genes are annotated with GO terms relating to immunity (red text). In both (B) and (C), the red bar indicates genes annotated with the COG term “posttranslational modification, protein turnover, and chaperones.” significance is calculated from DESeq2 (*** = adjusted *P* < 0.001, ** = adjusted *P* < 0.01, * = adjusted *P* < 0.05).

In agreement with previous bulk studies [14, 20, 65], our dataset demonstrated that, at the whole-organismal scale, immune system gene pathways identified by GO term analysis are differentially regulated under symbiosis. Genes belonging to differentially enriched immunity GO terms are most highly expressed in gastrodermal cells. In addition, expression of these genes is higher in gastrodermal cells from aposymbiotic (Fig. 6A) compared to symbiotic *O. arbuscula* (Fig. 6B). Taken together with the lack of observed differential expression between symbiotic states in the immune cell cluster (Fig. 5), these results suggest that symbiont-specific immune gene regulation occurs in gastrodermal cells rather than immune cells.

We identified the genes driving the clustering of Gastrodermis 1 and 2 cells by symbiotic state (see Fig. 4B, C). In Gastrodermis 1 and 2, we analyzed the distribution of the cells along each of the first 10 Principal Components (PCs) in the PCA dimensionality reduction used to generate the UMAPs. Gastrodermis 1 cells exhibited significant separation by symbiotic state along seven of the first 10 PCs (*P* < 0.05), and Gastrodermis 2 cells showed significant separation along eight of the first 10 PCs. In Gastrodermis 1, of the 89 annotated genes driving the distribution of the cells along the seven significant PCs, 29 were differentially expressed (adjusted *P* < 0.05), and 27 of the 89 genes were annotated with GO terms involved in immunity (Fig. 6B). Additionally, 17 genes were annotated with the COG term “posttranslational modification, protein turnover, and chaperones” (Fig. 6B). In Gastrodermis 2, of the 90 genes driving the distribution of the eight significant PCs 12 genes were differentially expressed (adjusted *P* < 0.05), 25 were annotated with immunity GO terms, and 14 were annotated with the COG term “posttranslational modification, protein turnover, and chaperones” (Fig. 6C). These results suggest that gastrodermal cells, rather than immune cells, differentially regulate immunity genes in symbiotic *O. arbuscula*.

### In two coral species, gastrodermal cells capable of symbiosis share functions relating to metabolism

To identify shared mechanisms by which specific types of gastrodermal cells host algae across coral species, we analyzed the overlap of differentially expressed orthologs (DEOs) between algal-hosting and non-algal hosting cells from our dataset (Gastrodermis 1, 2, 3, 4 and Algal-Hosting gastrodermal cells along the same cell fate trajectory in symbiotic and aposymbiotic *O. arbuscula*) to algal-hosting cells and other gastrodermal cells from an obligate coral species (*Xenia* sp. [59]). We identified 199 DEOs between *Xenia* algal-hosting and non-algal hosting gastrodermal cells (*Xenia*-sym-to-gastro) and 244 DEOs between *O. arbuscula* gastrodermal cells from symbiotic and aposymbiotic tissue (*Oculina*-sym-to-apo). We analyzed overlapping and unique DEOs between *Xenia*-sym-to-gastro and *O. arbuscula Oculina*-sym-to-apo: 73 DEOs were shared between the two datasets, 126 were unique to *Xenia*-sym-to-gastro, and 171 were unique to *Oculina*-sym-to-apo (Fig. S7). Using a Fisher Exact Test of BP GO terms, we probed functional enrichment of processes across each DEO group from the Venn diagrams. Across shared and *Oculina*-unique DEO groups, the most common GO terms were involved in metabolism, protein localization, and ribosomal function. Functions relating to cellular stress (e.g., “Stress-Activated MAPK Cascade”) and cell growth (e.g., “Regulation of Cell Growth”) were enriched in the *Oculina*-sym-to-apo-unique DEO set. Furthermore, the “Immune Response” GO term was enriched in the *Xenia*-sym-to-gastro-unique DEO set (Supplementary Dataset S3).

## Discussion

In this study, we have characterized how host immune gene expression appears to be modulated to facilitate endosymbiosis by comparing phenotypic metrics of symbiosis and profiling bacterial communities, whole-organism proteomics, and single-cell RNA sequencing (scRNA-seq) in symbiotic (with algal symbionts) and aposymbiotic (without algal symbionts) samples of the facultatively symbiotic stony coral *O. arbuscula*. This research suggests that cell type-specific compartmentalization of immune gene expression enables symbiotic hosts to simultaneously downregulate key immune pathways in algal-containing cells while maintaining whole organism immunity across other cell types. Furthermore, our results are consistent with emerging data indicating that the cnidarian-algal symbiosis is a balancing act between nutritional status and immunity [14, 20, 66]. Therefore, it appears that hosts must balance beneficial nutrient acquisition from photosynthetic algae with downregulation of the immune system within the same gastrodermal cells to allow for intracellular symbiont establishment and maintenance.

Whole-organism transcriptomic and proteomic research has yielded many insights into how symbiosis influences cnidarian host functions. For example, transcriptomic [14] and proteomic ([66] and this study) experiments have demonstrated how symbiotic state affects gene expression and protein abundance. Our whole-organism proteomic data across different symbiotic states under baseline conditions support results from previous experiments [14, 20, 65] demonstrating that differential regulation of the host immune system is required to maintain symbiosis. Specifically, our whole-organism proteomic analysis and bulk analysis of our single-cell data confirmed the pattern of differential regulation of immune system pathways under symbiosis in a facultatively symbiotic cnidarian, which has been previously reported for *O. arbuscula* [14], *E. pallida* [20], and *A. poculata* [27]. However, gene expression analyses from whole-organism studies may be confounded by differences in the proportions of cell types sampled and opposing RNA expression signals within these cell types [67]. Nevertheless, although the patterns between scRNA-seq and proteomic profiling observed here are consistent between aposymbiotic and symbiotic fragments, we still cannot determine whether the signature in the bulk proteomic data is driven by gastrodermal algal-hosting cells. Future work coupling scRNA-seq with single cell proteomic profiling [68] or immunohistochemistry would reveal more information about the localization of differentially enriched proteins across cell types.

scRNA-seq has been gaining traction in research on coral immune systems. One study [58] characterized two immune cell states with divergent expression patterns in obligate symbiotic stony coral *Stylaphora pistillata* adults. Additional studies [59, 69] described how LePin, a lectin involved in the phagocytic pathways of the cnidarian immune system, is required for symbiont establishment in algal-hosting cells of the soft coral *Xenia* sp. Cell state-specific regulation of the cnidarian immune system has also been proposed to occur in the sea anemone *E. pallida* via whole-larvae fluorescence imaging and transcriptomics [70]. For example, *MYD88*, encoding a protein that transduces extracellular signals into the host to induce the NF-κB pathway [71, 72], is reduced in *E. pallida* algal-hosting cells; this local immune modulation only occurs in symbiosis with compatible algae, thereby allowing host cells to sort and expel other non-symbiotic microbes [70]. Our scRNA-seq approach further highlights how changes to immune gene expression induced by hosting algal symbionts is compartmentalized in *O. arbuscula*, suggesting that cell state-specific immune modulation is key to the maintenance of a functional symbiosis within gastrodermal cells. Namely, we observed a downregulation of immune pathway genes in populations of symbiotic gastrodermal cells along the transcriptomic cell fate trajectory that terminates in algal-hosting cells, indicating that immune suppression is key to the maintenance of symbiosis in these cells. Although we were unable to confidently identify the gene for LePin in our data, we noted the clear involvement of immune gene regulation in driving transcriptomic differences in gastrodermal cells from symbiotic versus aposymbiotic tissue. Of note, these differentially expressed immunity genes included Heat-Shock Proteins (HSPs) and Cathepsins, which can be involved in the NF-κB pathway and protein chaperoning and are hypothesized to function in the regulation of symbiosis [73–76]. Induction of HSPs has been observed following thermal stress in symbiotic cnidarians, implicating the unfolded protein response in ensuring the stability of symbiosis [74, 77]. HSPs and Cathepsins have been previously shown to be markers for immune cells in the stony coral *S. pistillata* [58], but it remains to be determined whether this immune signature in gastrodermal cells is conserved in cnidarians engaged in obligate symbioses. Of the two gastrodermal cell types that we identified as displaying symbiosis-induced immune gene suppression, cells in the Gastrodermis 1 cluster exhibited stronger signals than those in Gastrodermis 2. This observation is most likely because Gastrodermis 1 contains more cells than Gastrodermis 2 (1,019 cells versus 837 cells, respectively), and therefore, the analysis of genes in Gastrodermis 1 had more statistical power.

Evidence for localized regulation of immune system pathways in algal-hosting gastrodermal cells has now been shown in a soft coral [69], a sea anemone [70], and a stony coral (present study). This compartmentalized immune system regulation likely allows for the maintenance of whole-organism immunity across other cell types, including cells in the Immune Cell cluster. We observed no differences in gene expression in the *O. arbuscula* Immune Cell cluster between symbiotic and aposymbiotic tissues. The maintenance of immune function across other cell types in *O. arbuscula*, both in and out of symbiosis, provides nuance to the proposed immunity-nutrient tradeoff that symbiotic cnidarians must balance: i.e., hosting algal symbionts within gastrodermal cells reduces algal-hosting cell immunity while not affecting whole-organism immunity.

The immune system has evolved in different ways across metazoans. For example, in the three-spined stickleback *Gasterosteus aculeatus*, eight distinct immune cell states were identified with distinct abundances and transcriptomic profiles across populations, demonstrating specialized and rapid evolution of compartmentalized immune regulation [78]. Here, we observe that “microevolution” of the immune system in *O. arbuscula* has occurred in three transcriptomically distinct immune cell states, indicating diversified immune cell function that is in agreement with other single-cell studies in stony coral [58]. “Microevolution” may also occur in specialized algal-hosting gastrodermal cells, allowing these gastrodermal cells to function as pseudo-immune cells without affecting immune gene expression in specialized immune cells. Because our data suggest that specialized immune cells are able to maintain constitutive immunity across symbiotic states, previous work in field-collected tropical corals showing differences in pathogen susceptibility by symbiotic state [23, 25] may be due to other factors (e.g., capacity for heterotrophy, efficiency of host-algal nutrient exchange, competition for nitrogen between host and algae) and therefore not be due to immune cell tradeoffs affected by symbiosis [26]. However, additional work focused on characterizing differences between cell type-specific immune regulation in obligate versus facultative cnidarians is necessary to validate this hypothesis. Our comparative analysis between *O. arbuscula* and *Xenia* sp. [59] algal-hosting and non-algal-hosting gastrodermal cells suggests that obligate and facultatively symbiotic cnidarians maintain their algal symbionts through different regulatory pathways, such as through classical immune response pathways in *Xenia* algal-hosting cells compared to cellular stress response pathways in *O. arbuscula* algal-hosting cells.

Our single-cell atlas also provides insights into carbon and nitrogen cycling within the gastrodermal cells of symbiotic and aposymbiotic *O. arbuscula*. We find that the gastrodermal cells that suppress immunity to allow for symbiosis are the same cells that undergo active nutrient cycling, most likely to regulate algal cell proliferation [11, 14, 79]. Previous work has proposed that increased nitrogen cycling and assimilation occurring in symbiotic host cells controls algal cell proliferation to maintain optimal nutrient exchange between host and symbiont, preventing parasitism of the symbiont on the host [11, 66]. In our data, the co-expression of lipid metabolism and nitrogen cycling genes within the same symbiotic cells in specific gastrodermal cell clusters supports recent studies emphasizing how lipid storage and glucose-dependent nitrogen competition are required for the stability of the cnidarian-algal relationship [12, 80]. Lipid bodies, which contain neutral lipids such as triacylglycerols, form in the algal-hosting gastrodermal cells of cnidarians [81]. The lipids that form the lipid bodies are, at least partially, derived from the endosymbiont [80–82]. In the nitrogen competition model, the metabolites and energy produced from host glycolysis of symbiont-derived glucose may be used by the host for amino acid synthesis, through which nitrogen species, such as ammonium, can be assimilated. Hosts can thus modulate the amount of ammonium translocated to the symbiont for proliferation [12]. In the present study, gastrodermal cells from aposymbiotic tissue exhibited lower expression of lipid metabolism and nitrogen cycling genes, which is likely driven by symbiotic cnidarians obtaining nutrients from symbiotic algae, whereas aposymbiotic *O. arbuscula* rely on heterotrophic sources to meet their metabolic needs [9, 83]. Thus, metabolic processing of energy and nitrogen likely occurs through distinct pathways in cnidarians that rely predominantly on heterotrophy (i.e., aposymbiotic corals) compared to those that rely predominantly on autotrophy (i.e., symbiotic corals) [66].

Although we observed no differences in the composition or diversity of the bacterial communities between aposymbiotic and symbiotic *O. arbuscula*, microbial partners may still play a role in nutrient cycling and possibly host immunity. Differences in bacterial composition by symbiotic state may not have been observed in our study because corals were housed in common garden conditions for at least three years. Future experiments with increased sample sizes collected *in situ* coupled with metabolomics or metatranscriptomics of the bacterial communities would more fully capture the functional variation of bacterial communities between symbiotic states.

Our scRNA-seq approach applied in a facultative coral provides a useful way to study the tradeoffs and cellular regulation associated with the cnidarian-algal symbiosis. Previous work using whole-organism experimental techniques suggested that immunity costs are associated with hosting algal symbionts [14, 20]. Here, we showcase how differences in cell states and gene expression can conflate patterns observed in whole-organism ‘omics studies and facilitate cell type-specific immune regulation to allow for endosymbiosis. Our description of highly specialized cell state-specific immune system regulation in a basal metazoan indicates that the immune costs of symbiosis previously suggested from whole organism studies might not be as costly as previously assumed. Future work should compare how compartmentalized immunity is modulated in different host-algal pairings in facultative versus obligate cnidarians and also determine how these cellular processes are shaped by changing environmental conditions.

## Supplementary Material

Accepted_Valadez-Ingersoll_supp_info_TrackChanges_wraf132

SuppDataset_1_rev2_wraf132

SuppDataset_2_rev2_wraf132

SuppDataset_3_rev2_wraf132

## Data Availability

Raw fastq files for 16S rRNA gene and single-cell RNA-seq data can be found on NCBI’s Short Read Archive Bioproject PRJNA1122932. The *O. arbuscula* genome is available on NCBI under the accession number GCA_964656845.1. All data and code for analyses can be found at https://github.com/mariaingersoll/Oculina_sc_manuscript.git.
