# Supplementary material for: Cell type-specific immune regulation under symbiosis in a facultatively symbiotic coral
Source: ISME J. 2025 Jun 26;19(1):wraf132. doi: 10.1093/ismejo/wraf132 (PMC12278269; doi:10.1093/ismejo/wraf132)
Supplement: Accepted_Valadez-Ingersoll_supp_info_TrackChanges_wraf132 [file accepted_valadez-ingersoll_supp_info_trackchanges_wraf132.docx]

***Supplementary Information***

**Cell type-specific immune regulation under symbiosis in a facultatively symbiotic coral**

Maria Valadez-Ingersoll^1^, Hanny E. Rivera^1, 2^, Jeric Da-Anoy^1^, Matthew R. Kanke^3^, Kelly Gomez-Campo^4, 5^, M. Isabel Martinez-Rugerio^4, 5^, Sebastian Metz^6^, Michael Sweet^6^, Julian Kwan^7^, Ryan Hekman^7^, Andrew Emili^1, 7, 8^, Thomas D. Gilmore^1^, Sarah W. Davies^1,*^

^1^Boston University, Department of Biology; Boston, MA, USA

^2^Ginkgo Bioworks, Boston, MA, USA

^3^Amgen Research, Research Bioimics, South San Francisco, CA, USA

^4^Pennsylvania State University, Department of Biology, State College, PA, USA

^5^Helmholtz Institute for Functional Marine Biodiversity (HIFMB), Oldenburg, Germany

^6^Aquatic Research Facility, Nature-based Solutions Research Centre, University of Derby, Derby, United Kingdom

^7^Department of Biochemistry, Boston University Chobanian & Avedisian School of Medicine, Boston, MA, USA

^8^Division of Oncological Sciences, Knight Cancer Institute, Oregon Health & Science University, Portland, OR, USA

*Corresponding author: Sarah Davies, Boston University, Department of Biology, 5 Cummington Mall, Boston, MA 02215; Phone: 617-353-8980; email: [daviessw@bu.edu](mailto:daviessw@bu.edu)

**Competing interests:** The authors declare no competing interests.

**Contents**

Supplementary Figures S1, S2, S3, S4, S5, S6, S7

Supplementary Tables 1, 2, 3

Supplementary Materials and Methods

**SUPPLEMENTARY FIGURES**

**
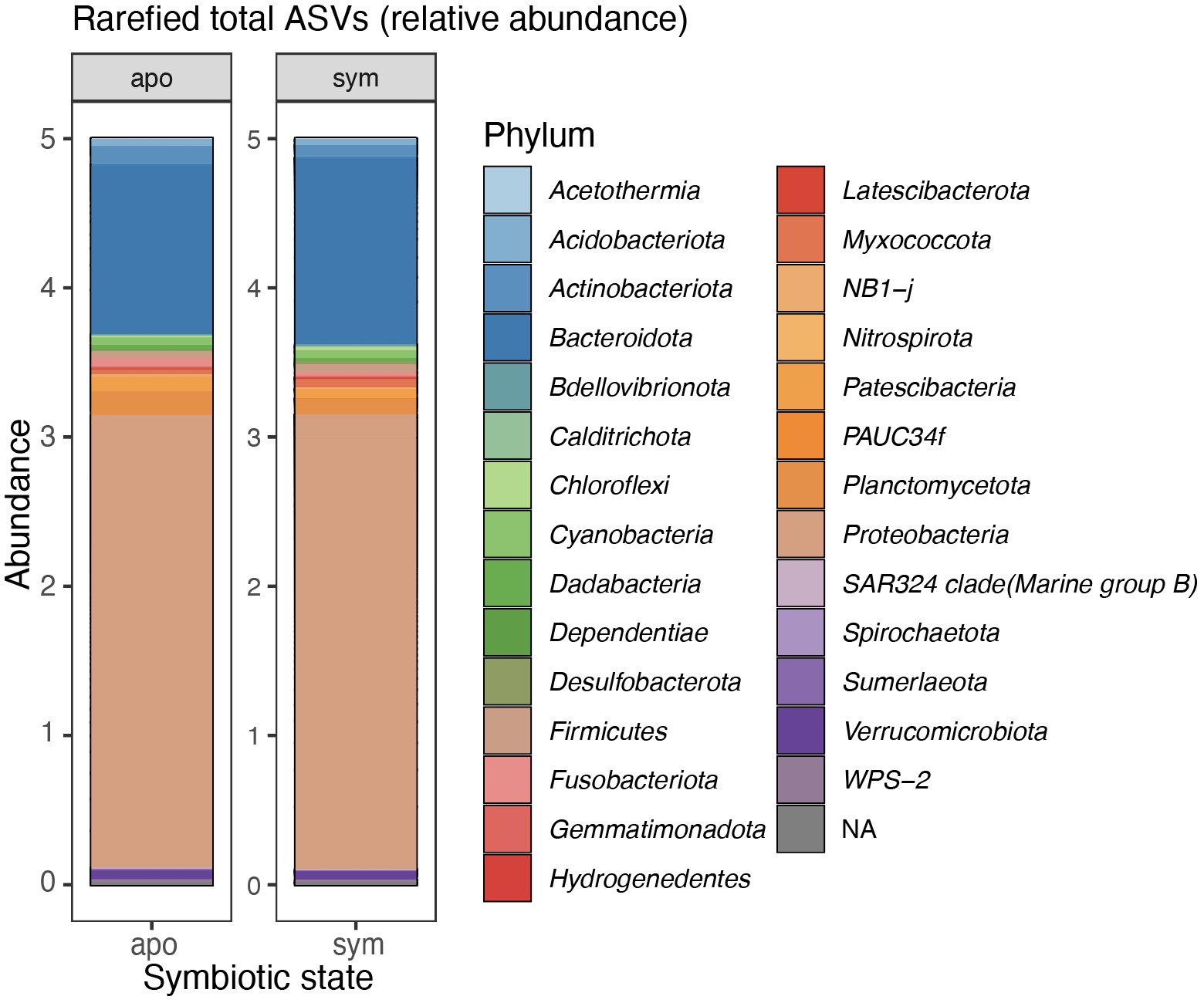
**

**Figure S1.** **Symbiotic and aposymbiotic *O. arbuscula* have similar bacterial communities.** Relative abundance of 16S rRNA gene ASVs from rarefied 16S rRNA gene sequencing reads across different bacterial phyla in symbiotic (sym) and aposymbiotic (apo) *O. arbuscula*.


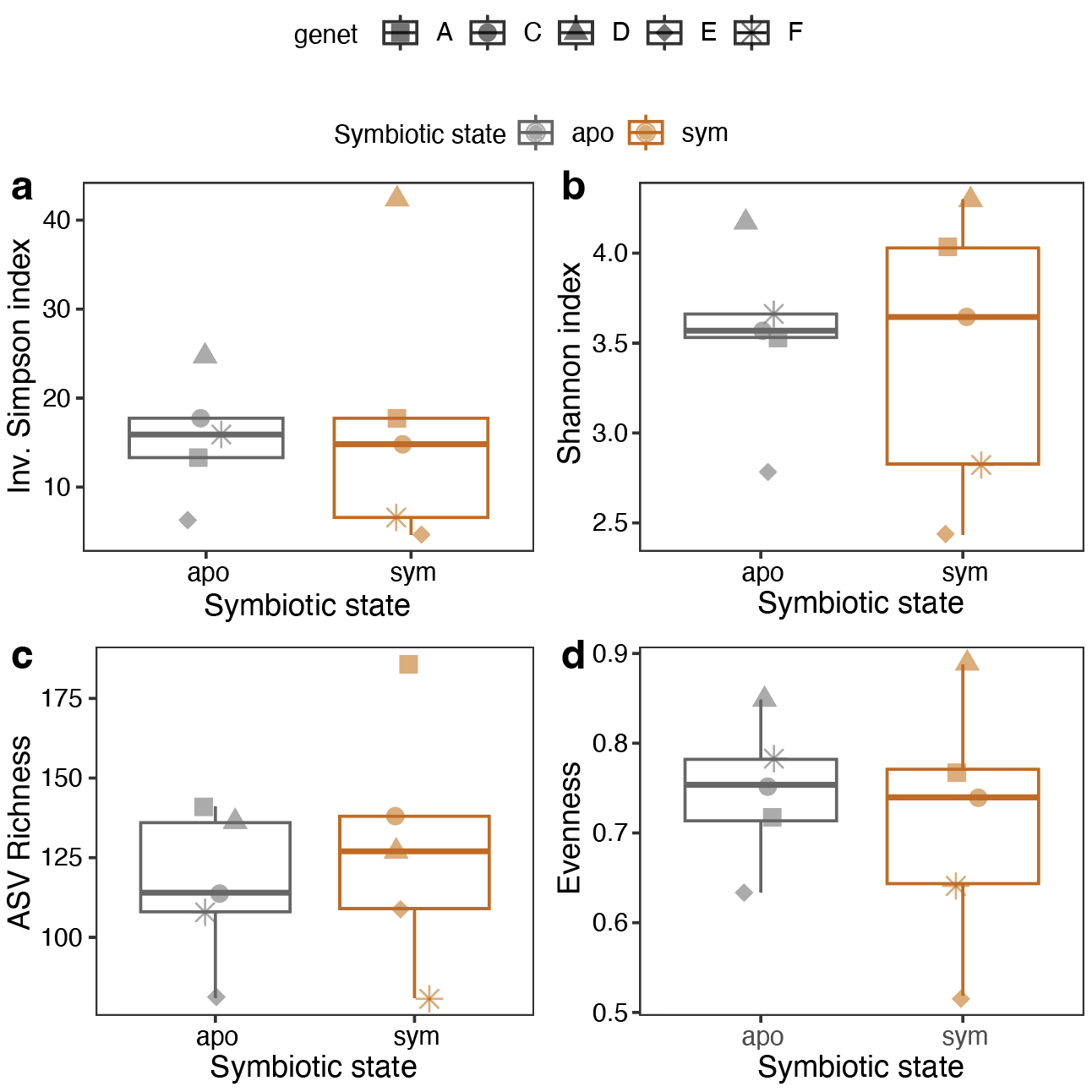


**Figure S2. No significant differences in bacterial diversity between symbiotic and aposymbiotic *O. arbuscula.*** 16S rRNA gene sequencing data showing alpha diversity (**a** Simpson Diversity; **b** Shannon Diversity, **c** Observed Species Richness from rarefied reads; **d** Evenness) between symbiotic (sym) and aposymbiotic (apo) *O. arbuscula*. Box plots show the median and the 1st and 3rd quartiles (hinges). Whiskers extend to the maximum and minimum points if they fall within 1.5 x the Interquartile Range (if no whiskers are drawn, points outside of the box are outliers). Each point is the alpha diversity metric of one individual (with the shapes representing genets).

**
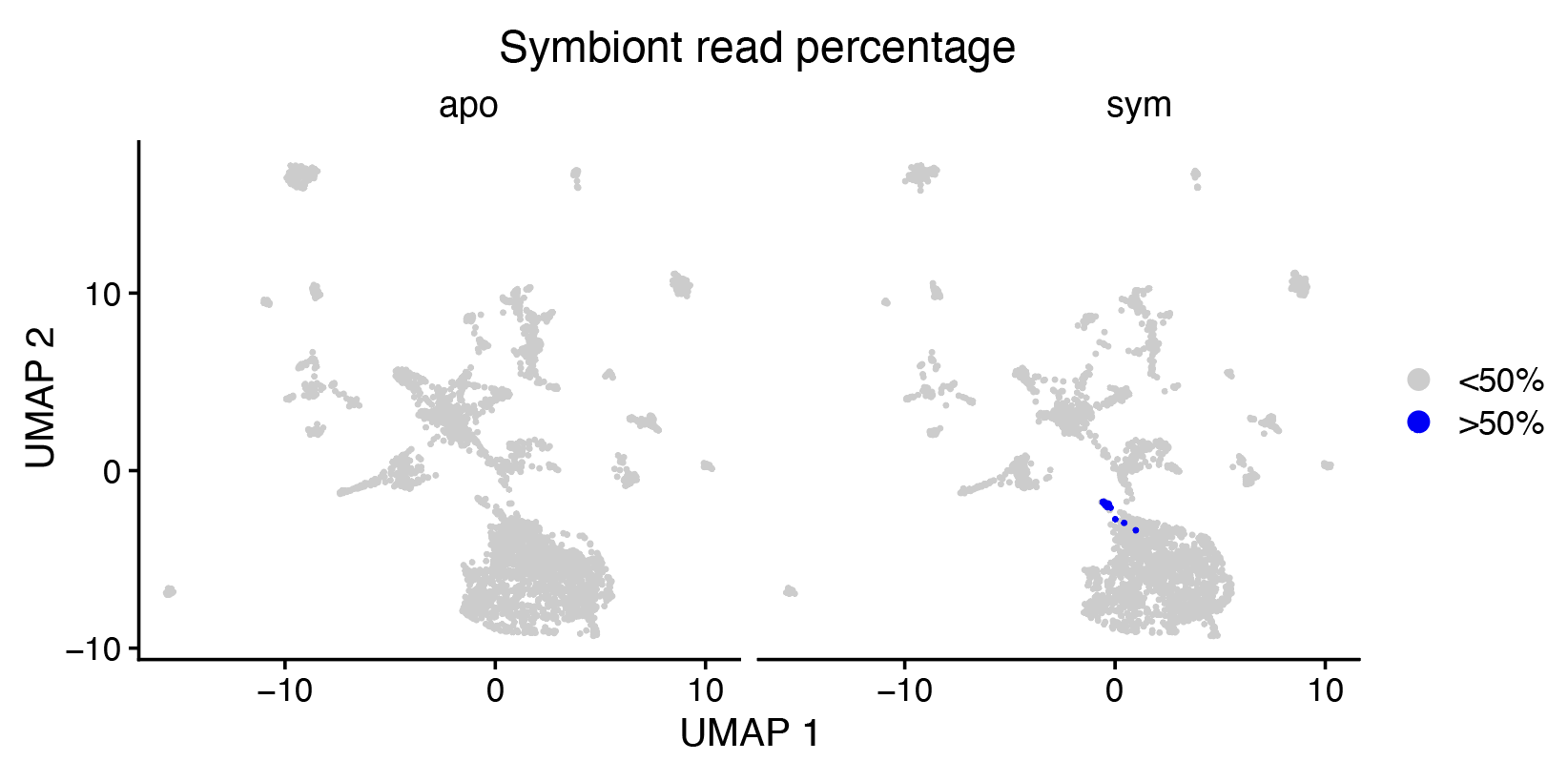
**

**Figure S3. Algal-Hosting cells identified in symbiotic *O. arbuscula.*** Cells in which over 50% of the reads correspond to *B. psygmophilum* genes are highlighted in blue. 45 cells were identified, 42 of which were found in one cell cluster in the symbiotic sample, deemed the Algal-Hosting gastrodermal cell cluster.

**
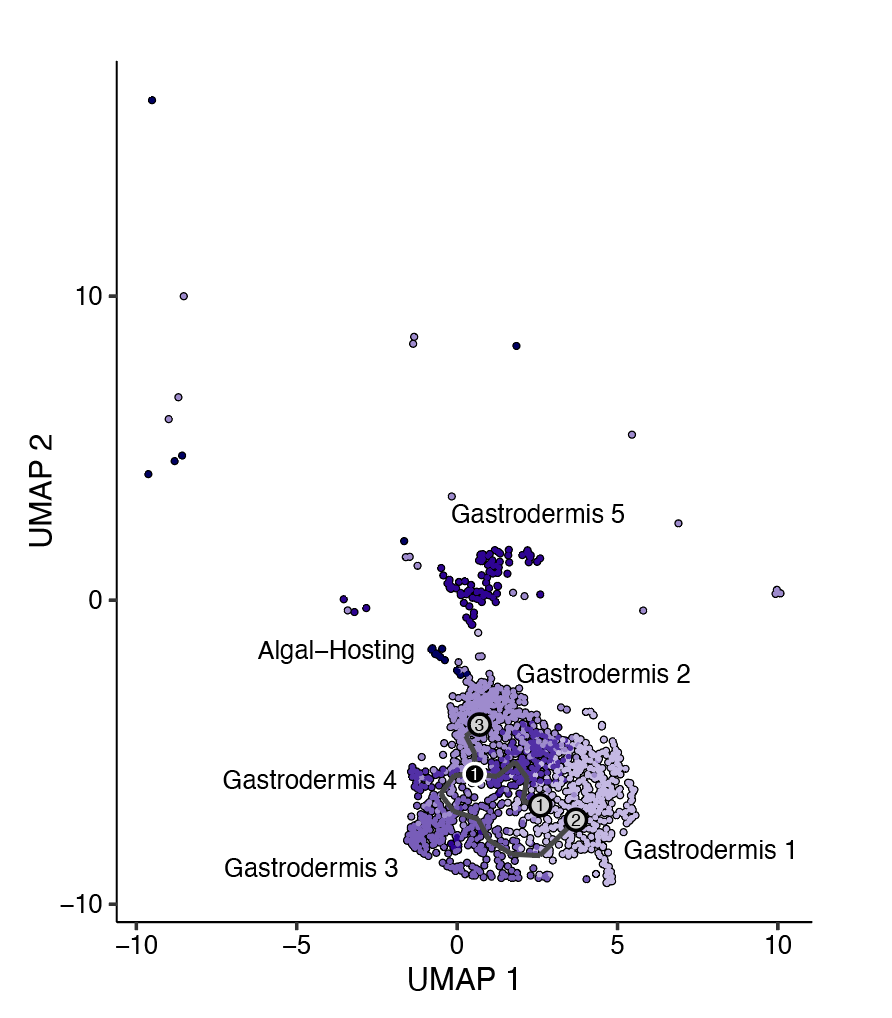
**

**Figure S4. Gastrodermis cells from aposymbiotic *O. arbuscula* have three outcomes.** UMAP projection of Gastrodermis 1, 2, 3, 4, 5, and Algal-Hosting cells from aposymbiotic *O. arbuscula* overlayed with a graph of cell trajectories. The three transcriptomic outcomes (fates) are denoted by grey circles with black numbers. Branch nodes are denoted by black circles with white numbers.

**
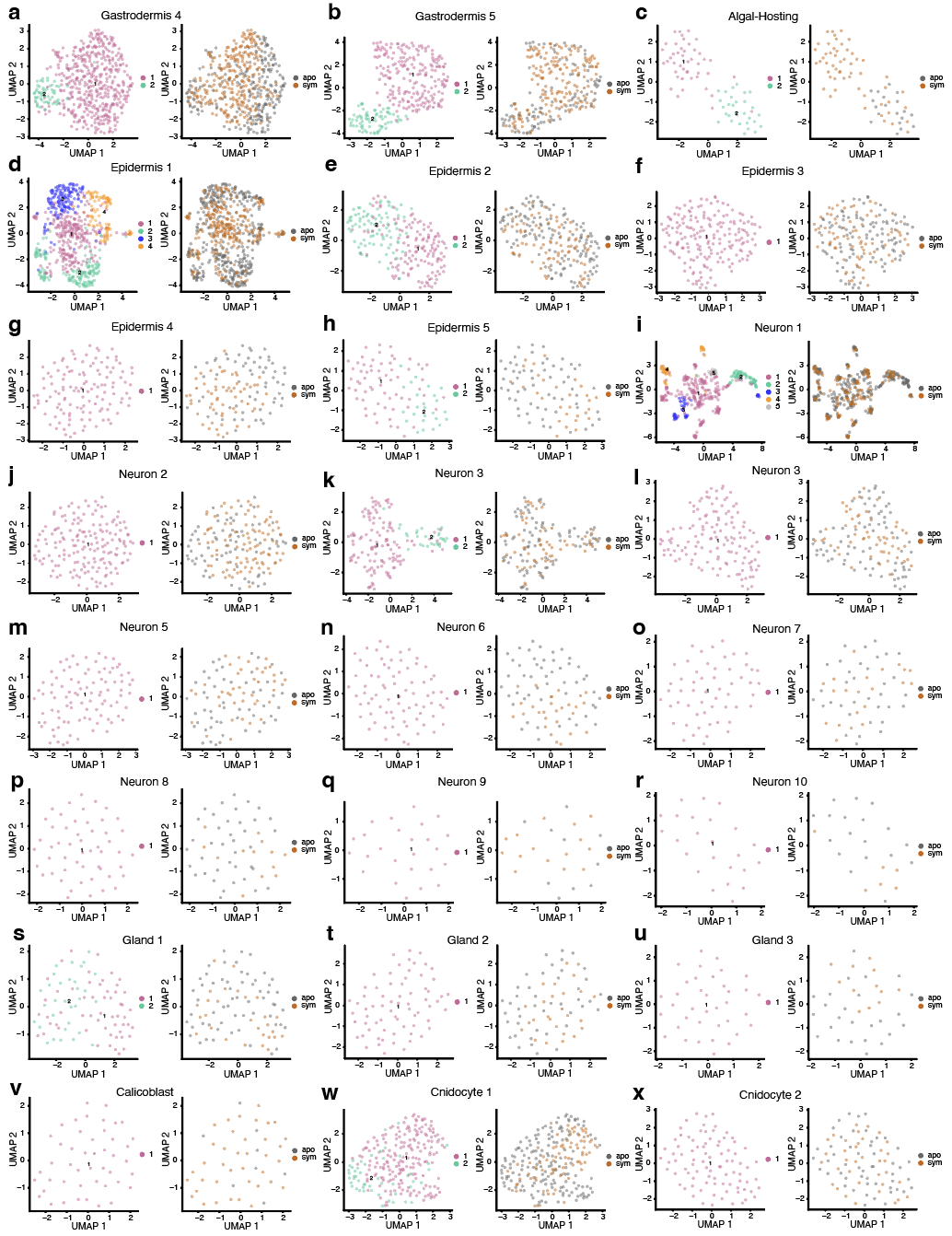
**

**Figure S5. Separation of cell clusters between subclusters and symbiotic states within different cell types.** UMAP projections of subsetted and reclustered cells from Gastrodermis 4 (**a**), Gastrodermis 5 (**b**), Algal-Hosting (**c**), Epidermis 1 (**d**), Epidermis 2 (**e**), Epidermis 3 (**f**), Epidermis 4 (**g**), Epidermis 5 (**h**), Neuron 1 (**i**), Neuron 2 (**j**), Neuron 3 (**k**), Neuron 4 (**l**), Neuron 5 (**m**), Neuron 6 (**n**), Neuron 7 (**o**), Neuron 8 (**p**), Neuron 9 (**q**), Neuron 10 (**r**), Gland 1 (**s**), Gland 2 (**t**), Gland 3 (**u**), Calicoblast (**v**), Cnidocyte 1 (**w**), Cnidocyte 2 (**x**). For each cell type, subclusters are color-coded in the left panel, and the symbiotic state of each cell is colored in the right panel.

**
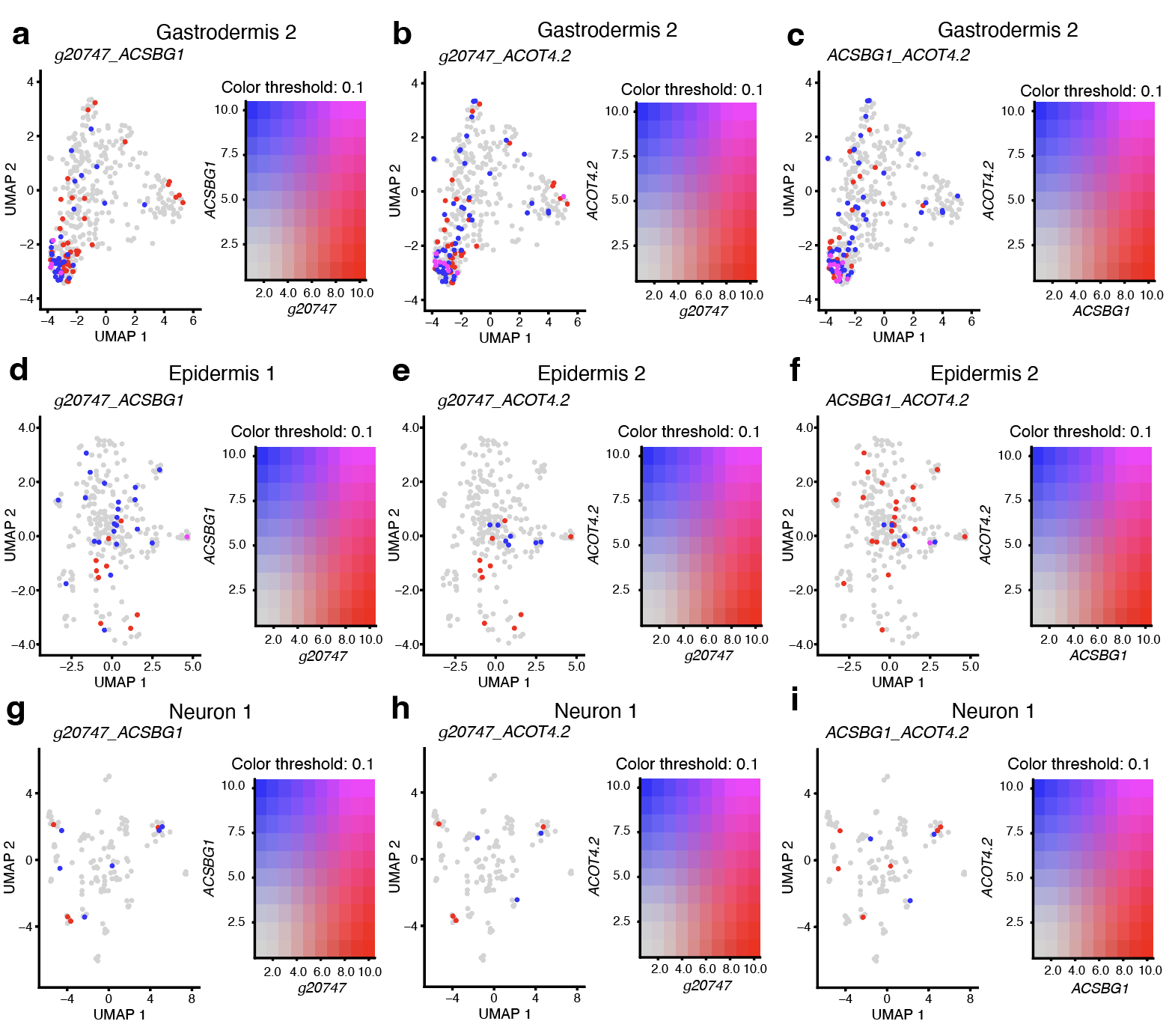
**

**Figure S6. Nutrient cycling genes of interest show little co-expression in Epidermis 1 and Neuron 1 cell clusters as compared to Gastrodermis 1 and 2.** Co-expression plots of *g20747* and *ACSBG1*, *g20747* and *ACOT4.2*, and *ACSBG1* and *ACOT4.2* across Gastrodermis 2 cells (**a, b, c**), Epidermis 1 cells (**d, e, f**), and Neuron 1 cells (**g, h, i**) from symbiotic *O. arbuscula.* The coloration of each cell represents the per-cell mean expression value of each gene scaled to a maximum value of 10.

**
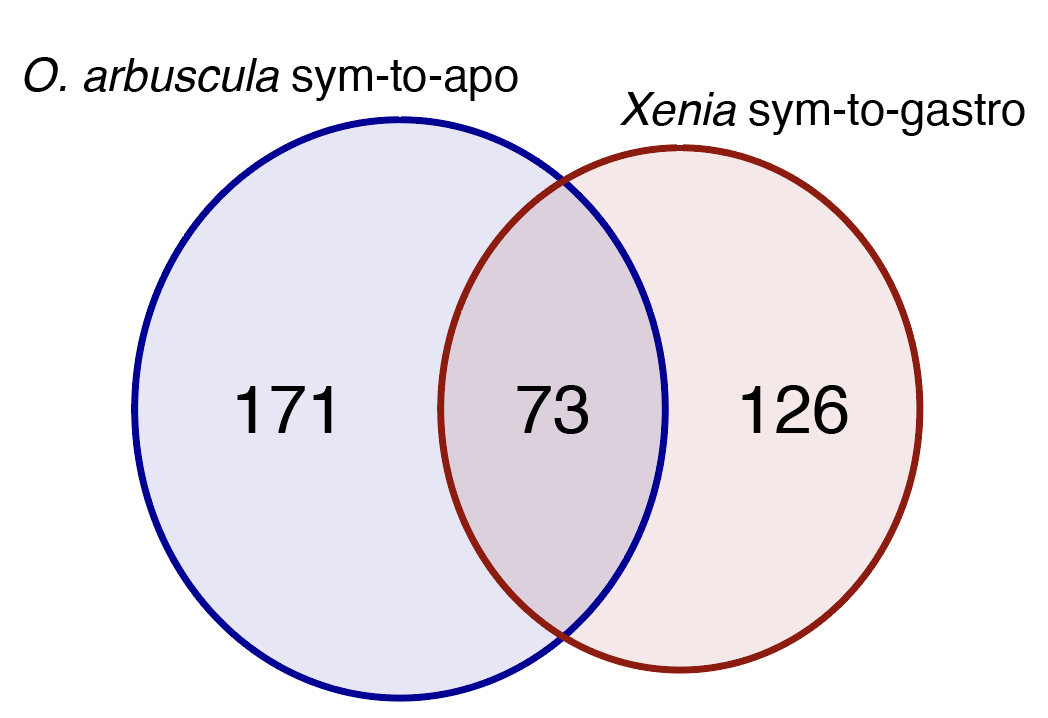
**

**Figure S7.** **Differentially expressed orthologs between *Xenia* and *O. arbuscula* algal-hosting and non-algal-hosting gastrodermal cells.** Overlap of differentially expressed orthologs (DEOs) between *O. arbuscula* Gastrodermis 1, 2, 3, 4, and Algal-Hosting gastrodermal cells from symbiotic and aposymbiotic tissue (*Oculina*-sym-to-apo) and *Xenia* algal-hosting and non-algal hosting gastrodermal cells (*Xenia*-sym-to-gastro). *Xenia* data from (32).

**SUPPLEMENTARY TABLES**

**Table S1.** Biological Process GO terms involved in immunity that are differentially enriched between symbiotic and aposymbiotic *O. arbuscula* based on bulk analysis of scRNA-seq data.

| **Term** | **Name** | **adjusted *P* value** |
| --- | --- | --- |
| **GO:0034123** | **positive regulation of toll-like receptor signaling pathway** | **0.03040438** |
| **GO:0034121** | **regulation of toll-like receptor signaling pathway** | **0.06616590** |
| **GO:0032717** | **negative regulation of interleukin-8 production** | **0.03582820** |
| **GO:0032677**  **GO:0032757** | **regulation of interleukin-8 production** | **0.05871839** |
| **GO:0038111** | **interleukin-7-mediated signaling pathway** | **0.06130530** |
| **GO:0032663** | **regulation of interleukin-2 production** | **0.09174735** |
| **GO:0070670**  **GO:0071353** | **response to interleukin-4** | **0.09183266** |
| **GO:0043123** | **positive regulation of I-kappaB kinase/NF-kappaB signaling** | **0.06811408** |
| **GO:1901222** | **regulation of NIK/NF-kappaB signaling** | **0.08081779** |
| **GO:0071560**  **GO:0071559** | **response to transforming growth factor beta** | **0.06831721** |
| **GO:0030511** | **positive regulation of transforming growth factor beta receptor signaling pathway** | **0.09833854** |

**Table S2.** Resolutions used to define subclusters within each cell cluster of the full dataset.

| **Cluster** | **Resolution** |
| --- | --- |
| Gastrodermis 1 | 0.25 |
| Gastrodermis 2 | 0.25 |
| Gastrodermis 3 | 0.30 |
| Gastrodermis 4 | 0.30 |
| Gastrodermis 5 | 0.30 |
| Algal-Hosting | 0.30 |
| Epidermis 1 | 0.50 |
| Epidermis 2 | 0.30 |
| Epidermis 3 | 0.30 |
| Epidermis 4 | 0.30 |
| Epidermis 5 | 0.30 |
| Neuron 1 | 0.30 |
| Neuron 2 | 0.30 |
| Neuron 3 | 0.30 |
| Neuron 4 | 0.30 |
| Neuron 5 | 0.50 |
| Neuron 6 | 0.50 |
| Neuron 7 | 0.50 |
| Neuron 8 | 0.50 |
| Neuron 9 | 0.50 |
| Neuron 10 | 0.50 |
| Gland 1 | 0.30 |
| Gland 2 | 0.30 |
| Gland 3 | 0.50 |
| Calicoblast | 0.50 |
| Cnidocyte 1 | 0.50 |
| Cnidocyte 2 | 0.50 |
| Immune Cell | 0.40 |

**Table S3.** Raw and filtered read counts for samples used in 16S rRNA gene profiling (sym=symbiotic and apo=aposymbiotic).

| **Sample** | **Sym State** | **Raw** | **Filtered** |
| --- | --- | --- | --- |
| A13 | Sym | 21357 | 20430 |
| A8 | Apo | 21478 | 20255 |
| F8 | Apo | 10073 | 9138 |
| F1 | Sym | 13139 | 12627 |
| D7 | Sym | 14345 | 13561 |
| D6 | Apo | 21389 | 20513 |
| E8 | Apo | 13400 | 12994 |
| E4 | Sym | 65019 | 63410 |
| C6 | Apo | 14825 | 14102 |
| C9 | Sym | 13305 | 11219 |

**SUPPLEMENTARY MATERIALS AND METHODS**

Detailed information on the sample preparation, bioinformatic analyses, and R packages necessary to complete the experiments outlined in the Materials and Methods section of the manuscript *Cell type-specific immune regulation under symbiosis in a facultatively symbiotic coral*.

##

## **Coral husbandry and manipulation of symbiotic state**

Symbiotic (in symbiosis with *Breviolum psygmophilum*) (1) colonies from seven genetic backgrounds (genets A-G) of *Oculina arbuscula* were collected at Radio Island Jetty, North Carolina (34˚ 42.520’ N, 76˚ 40.796’ W) in May 2018 under NC Division of Marine Fisheries Permit #1627488. These colonies have been maintained at Boston University in common garden aquaria (25 °C (± 0.17, SD), 34.6 PSU (± 0.76, SD), pH 8.0 (± 0.08, SD)) since May, 2018. One subset of aposymbiotic *O. arbuscula* branches was generated via menthol bleaching in Spring 2021 (symbiont counts, 16S rRNA gene sequencing, proteomics) and a second subset was generated in Spring 2022 (scRNAseq). Briefly, branches were incubated in 0.58 mM menthol in seawater under common garden light conditions. The menthol solution was replaced every 24 h for of two weeks. After aposymbiotic status was confirmed by a lack of symbiont autofluorescence under fluorescence microscopy (Leica M165 FC), aposymbiotic branches were transferred back to common garden aquaria and fragments were maintained for at least two months of recovery prior to physiological and multiomic profiling.

**Tissue removal for symbiont cell quantification**

To measure symbiont cell densities hosted in branches of symbiotic and aposymbiotic *O. arbuscula*, branches of approximately 2.5 cm in length were fragmented from symbiotic (N=10) and aposymbiotic (N=10) samples from genets A, C, D, E, and F, with at least one sample per genet for each symbiotic state. Tissue was removed via airbrushing into 0.2 μM-filtered Artificial Seawater (Instant Ocean). Total volume was noted, the resulting tissue slurry was homogenized, and an aliquot was subsampled for symbiont quantification using a hemocytometer. Skeleton surface area was measured using an Einscan-SE scanner and MeshLab software, and symbiont cells were normalized to surface area. Statistical differences between symbiotic and aposymbiotic *O. arbuscula* fragments were calculated using the Kruskal-Wallis rank sum test in Rstudio v4.3.0, as assumptions of normality were not met.

**Coral spectroscopic determinations**

The light absorption capacity of symbiotic and aposymbiotic *O. arbuscula* was compared using reflectance (R) and absorptance (A) (2–4). Coral reflectance (R), i.e., the fraction of light reflected by the sample, was measured between 400-750 nm in intact coral fragments using a miniature spectroradiometer (Flame-T-UV-Vis, Ocean Optics Inc.). Briefly, samples were placed in a black container filled with filtered seawater and illuminated with homogeneous diffuse light by positioning a semi-sphere with an internal reflecting coating (barium oxide BaO) above the sample, and a ring of LEDs and halogen lamps directed upwards into the reflective coating. The reflected light was collected by a 2 mm diameter fiber-optic placed 1 cm above the surface of the sample at an angle of 45°. Reflectance was expressed as the ratio of the measurement from the tissue surface relative to the reflectance of a coral reference (a bleached *O. arbuscula* skeleton cleaned with commercial Hydrochloric Acid HCl). The coral absorptance (A), which describes the fraction of incident light absorbed by the coral tissue, was calculated from the reflectance spectra as A = 1 – R (2,4). The absorptance peak of chlorophyll a (Chl a) at 675 nm was calculated as A_675_ = 1 – R_675_, assuming that transmission through the skeleton of the samples is negligible.

**Microbiome profiling**

To identify bacterial communities associated with symbiotic and aposymbiotic fragments, one symbiotic and one aposymbiotic fragment from genotypes A, C, D, E, and F were flash frozen, and one polyp from each fragment was preserved in ethanol for 16S rRNA gene sequencing (N=10). Libraries were generated using a series of PCR amplifications for the V4/V5 region of the bacterial 16S rRNA gene (5,6) as follows: 95°C for 40 sec, 58°C for 120 sec, and 72°C for 60 sec for 32 cycles, with a final elongation step of 72°C for 5 min. PCR products were purified using GeneJET PCR Purification kits (ThermoFisher) and eluted in 30 µl. Each PCR product was barcoded via five PCR cycles and visualized on a 1% agarose gel to assess relative concentrations. Five negative controls using water were prepared and later used to remove contaminating sequences. Samples were pooled in equal concentrations, gel-extracted, and submitted for paired-end 250 bp sequencing on a Miseq (Illumina) at Tufts University Core Facility.

16S rRNA gene primers were removed from raw reads using cutadapt (7). DADA2 v1.28.0 (8), quality filtering was conducted, and 1,232 sequence variants (ASVs) were inferred. Taxonomy was assigned at 100% sequence identity using the *Silva* v. 138.1 database (9). ASVs matching mitochondria, chloroplasts, or non-bacterial kingdoms were removed (83 ASVs removed) and 20 ASVs were removed based on negative controls as contaminants (decontam v1.2.0; (10)). Cleaned counts were rarefied to 8,298 using vegan v2.6-4 (11) and trimmed using MCMC.OTU v1.0.10 (12) to remove ASVs in less than 0.02% of counts, resulting in 661 ASVs across samples. The composition of bacterial communities across symbiotic state were compared via alpha diversity (Shannon index, Simpson’s index, ASV richness, and evenness) using phyloseq v1.44.0 (function *estimate_richness* (13)) and beta diversity was assessed using a PCoA on Bray-Curtis dissimilarity with a PERMANOVA in the vegan package (v2.6-4 (11)). Alpha diversity metrics were compared using linear mixed effects models ((package lme4; v1.1-35.3 (14)) with symbiotic state as the predictor and a random effect of coral genotype. The effect of symbiotic state on beta diversity was assessed using the function *betadisper* (vegan package; v2.6-4 (11)). DESeq2 v1.40.2 (15) then explored differentially abundant ASVs to ensure that subtle differences in specific taxa were not overlooked.

***Oculina arbuscula* genome assembly and annotation**

A chromosome-level genome assembly of *O. arbuscula* was recently released and is available from NCBI under the accession number GCA_964656845.1. This genome was generated by the Aquatic Symbiosis Genomics Project (https://www.aquaticsymbiosisgenomics.org) using ~25× coverage PacBio HiFi reads and Arima Genomics Hi-C data. The initial assembly was produced with Hifiasm (16) in Hi-C integrated assembly mode. Scaffolding based on Hi-C contact maps was performed using YaHS (17). The mitochondrial genome was assembled with MitoHiFi v2.2 (18), and each haplotype assembly was manually curated using TreeVal (19). Chromosome-scale scaffolds confirmed by Hi-C contact maps were named according to their size.

For structural gene annotation, the genome was first soft-masked using RepeatModeler2 v2.0.5 (20) with the -LTRStruct module enabled. Transcriptomic data were retrieved from GenBank using VARUS (21) and aligned to the genome with HISAT2 v2.2.1 (22). The soft-masked genome, aligned RNA-seq reads, and a Scleractinia protein dataset containing over 1.2 million sequences were used for gene prediction with BRAKER3 (23). The resulting gene models were further refined through a second BRAKER3 run, incorporating a curated set of proteins from published Robust clade coral genomes. The final predicted proteome was functionally annotated using EggNOG-mapper v2.1.12 (24), and the completeness of the annotation was assessed using BUSCO v5.8.2 (25) against the Cnidaria_odb12 reference dataset.

**Proteomic profiling**

Mass spectroscopy (MS) was used to identify differentially enriched proteins from total protein isolated from fragments of five symbiotic genets (Ax2, C, E, and F) and three aposymbiotic genets (C, E, and F). As described previously (26), each fragment (approximately 1 cm x 1 cm) was washed in PBS then crushed and incubated in 1X AT Lysis buffer with proteinase inhibitors (10 mM HEPES pH 7.9, 1 mM EDTA, I  mM EGTA, 20% (w/v) glycerol, 1% w/v Triton X-100, 20 mM NaF, 1 mM Na_4_P_2_O_7_·10H_2_O, 1 mM dithiothreitol, 1 mM phenylmethylsulfonyl fluoride, 1 μg/ml leupeptin, 1 μg/ml pepstatin A, 10 μg/ml aprotinin) for 1 h at 4 ℃ on an orbital shaker with occasional vortexing. The lysate was clarified by centrifugation at 13,000 rpm for 15 min at 4 ℃, and the supernatant was stored at -80 ℃ prior to analysis by MS.

For MS, tryptic peptide mixtures were analyzed by nano-scale high-performance liquid chromatography (Proxeon EASY-Nano system, Thermo Fisher Scientific) coupled with online nanoelectrospray ionization tandem MS (Q-Exactive HF-X mass spectrometer; Thermo Fisher Scientific). Briefly, samples were loaded into the system with aqueous 0.1% (v/v) formic acid via a trap column (75 μm i.d. × 2 cm, Acclaim PepMap100 C18 3 μm, 100 Å, Thermo Fisher Scientific) and peptides were resolved over an Easy-Spray analytical column (50 cm × 75 μm ID, PepMap RSLC C18, Thermo Fisher Scientific) by an increasingly mobile phase B comprising 2% acetonitrile and 0.1% formic acid, whereas organic phase B consisted of 80% acetonitrile and 0.1% formic acid. Reverse phase separation was performed over 120 min at a flow rate of 300 μl/min. Eluted peptides were ionized directly into the mass spectrometer using a nanospray ion source. The mass spectrometer was operated in positive ion mode with a capillary temperature of 300 ℃ and a potential of 2,100 V applied to the frit. Tandem mass spectrometry (MS/MS) was performed using high-energy collision-induced dissociation, and 10 MS/MS data-dependent scans (45,000 resolution) were acquired in profile mode alongside each profile mode full-scan mass spectra (120,000 resolution), as previously described (27). For MS scans, the automatic gain control (AGC) was set at 1 × 10^6^ ions with a maximum fill time of 60 ms. MS/MS scans had an AGC of 3 × 10^4^, with a maximum injection time of 80 msec, activation time of 0.1 msec, and 33% normalized collision energy. To prevent repeated selection of peptides for MS/MS, a dynamic exclusion list was activated to exclude all fragmented ions for 60 sec.

For protein identification and analysis, data files (RAW format) were searched using the standard workflow of MaxQuant version 2.4 (<http://www.maxquant.org/>) under standard settings using the *O. arbuscula* genome. The parameters included two missed trypsin cleavage sites, fixed carbamidomethylation of cysteine, variable methionine oxidation, protein N-terminal acetylation, and phosphorylation of STY residues. For the first search, precursor ion tolerances were set at 20 ppm, and for the second search, they were set to 4.5 ppm. The MS/MS peaks were de-isotoped and searched using a 20-ppm mass tolerance. A stringent false discovery rate (FDR) threshold of 1% was used to filter candidate peptides and protein identifications. The searched intensity data were filtered, normalized, and clustered using Omics Notebook (28). Filtering was performed to remove any proteins not identified in at least 70% of samples, with 2,543 proteins passing the filter. After filtering, both datasets showed low levels of sparsity, and no missing value imputation was performed. The LIMMA R package was used for LOESS normalization and differential abundance analysis (29). Differential analysis of proteomic profiles between aposymbiotic and symbiotic samples was based on a moderated t-test (28). Proteins were considered differentially abundant if they had a Bonferroni adjusted *P* value < 0.1. Raw intensity counts were normalized using the rlog transformation function in DESeq2 (15) and visualized using a Principal Component Analysis (PCA) with the package vegan v2.6-4 (11). The effects of symbiotic state and genet were assessed using PERMANOVA with the adonis2 function in vegan v2.6-4 (11).

Predicted peptides of *O. arbuscula* were searched against the human proteome v.11.5 from the STRING v.11 database (30) with an e-value cut-off of 1x10^-5^. Protein–protein interactions of select differentially expressed proteins (FDR < 0.1) were retrieved from the STRING v.11 database (30). Interaction networks were visualized using Cytoscape v.3.7.2 (31).

**Single-cell RNA sequencing**

To create single-cell libraries, live cells from one symbiotic (F1) and one aposymbiotic (F8) branch were sampled from the same genet for single-cell isolation and 10X cDNA sequencing, using a previously verified protocol for coral cell isolation (32). As described on protocols.io at <http://dx.doi.org/10.17504/protocols.io.rm7vzkx72vx1/v1>, each fragment of approximately 1.5 cm in length was placed into 10 ml of ice-cold calcium-free artificial seawater (0.2-micron filter-sterilized). The fragment was then moved to 10 ml of ice-cold cell-isolation media (3.3x PBS, 2% heat-inactivated fetal bovine serum, 20 mM HEPES buffer in deionized H_2_O). Using RNase-free forceps, tissue was mechanically scraped from the skeleton until no visible tissue remained (<10 min). On ice, the cell-slurry was filtered through a 70-μm filter, then through two 40-μm filters. This process was repeated for each sample. 1 ml of filtered cell suspension was centrifuged at 4℃ for 10 min at 300 x g, and the pellet was resuspended in 100 μl of cell-isolation media with 0.2 U/μl Protector RNase Inhibitor. To maintain viability, cells were not sorted prior to processing. However, because we did not perform cell sorting through a method such as Fluorescence-activated Cell Sorting, multiplets may be present in our dataset. Cell isolation samples were analyzed by the Boston University Single Cell Sequencing Core Facility where cell counts and viability were quantified by 0.04% Trypan Blue staining and counting with a hemocytometer. Symbiotic branch F1 had a concentration of 2,625 cells/μl and a viability of 81.6%. Aposymbiotic branch F8 had a concentration of 3,312.5 cells/μl and a viability of 86.4%. These viability values are largely in line with other single cell preparation methods reported in the literature (viability threshold of 80%, *Nematostella vectensis* (33,34)).

cDNA for each sample was generated following the 10X Genomics Chromium Single Cell 3’v3 protocols, and quality was assessed via Bioanalyzer High Sensitivity DNA analysis. Samples were pooled in equimolar concentration, and the library was sequenced on a NextSeq 2000 (Illumina, P3 100 kit) to obtain 50,000 reads per cell. 10X Genomics CellRanger (version 7.2.0 (35)) processed the sequencing reads, which were then aligned to concatenated genomes of *O. arbuscula* (host) and the algal symbiont *B. psygmophilum* (36). Reads aligning confidently to the host and symbiont references were used to generate CellRanger output files. To create cell profiles for *O. arbuscula* and assign cell state identities, host reads from symbiotic and aposymbiotic samples were analyzed using Seurat (v.5.0.2) (37) in Rstudio (v.4.2.3). First, doublets were detected and removed using scDblFinder (v.1.20.2) on each sample independently (38). Next, genes expressed in fewer than three cells were discarded. Cells expressing fewer than 200 genes were removed, as were cells expressing greater than 3,000 genes. No duplicated barcodes were detected. Mitochondrial reads could not be removed, as the reference genomes/transcriptomes do not have annotated mitochondrial genomes. Using all 20,719 host genes with non-zero read counts, datasets were first independently log-normalized, expression values of these genes were scaled, and a PCA was performed. To more accurately identify cell clusters across both symbiotic states, the datasets were integrated using the Canonical Correlation Analysis (CCA) Integration method on the PCA reduction (nearest neighbors parameters). Clusters were identified using 30 dimensions and a resolution of 0.55 and were visualized using Uniform Manifold Approximation and Projection (UMAP). Marker genes were identified using FindAllMarkers (Wilcoxin Rank Sum Test; log_2_foldchange threshold of 0.5) and cell types were informed by gene annotations and marker gene comparisons to other cnidarian single-cell datasets (e.g., *S. pistallata* and *Xenia* sp.) (**Supplementary Dataset 1**) (39,40). Expression patterns of top marker genes were assigned to cell clusters using violin plots, bubble plots, and visualization of gene expression in individual cells within the UMAP. 28 cell clusters across seven cell types were identified in the UMAP. Lastly, to assess the broad transcriptomic differences between each cell cluster, the top 10 genes enriched in each cell cluster (using the average log_2_foldchange from FindAllMarkers) were identified.

To identify cells containing algal symbionts, we identified cells in which over 50% of the total reads corresponded to *B. psygmophilum* genes and then visualized these cells on the UMAP.

To determine how symbiosis alters enrichment of gene pathways at the whole-organism level, and how this differential enrichment is reflected in specific cell types, we performed Mann-Whitney U Gene Ontology (GO) term enrichment analysis (41). Significantly enriched GO terms within the ‘Biological Process’ (BP) GO division were defined with a false-discovery rate (FDR) of <0.1. Significantly over- and under-represented GO terms relating explicitly to the NF-κB pathway, Toll-Like Receptor signaling, Interleukins, and Transforming Growth Factor β were identified (**Table S1**). Cells in which over 4.5% of the total reads corresponded to genes annotated with the GO terms of interest were visualized on the UMAP of all cells from symbiotic and aposymbiotic *O. arbuscula* samples.

To compare expression profiles of specific cell states between symbiotic and aposymbiotic samples, each cell cluster was independently reclustered, and the effect of symbiotic state on gene expression in each cell state was analyzed. First, the cell cluster of interest (*e.g.,* Immune Cell, Gastrodermis 1) was subsetted from the full dataset, and new variable features were identified. Data were then rescaled, and dimensionality reduction was re-run (using the first 30 dimensions for all clusters, except for Neuron 9 and Neuron 10, for which 20 dimensions were used). Subclusters were identified with resolutions between 0.25-0.5 (**Table** **S2**). The distribution of cells across the subclusters according to the symbiotic state was visualized using UMAPs. Differentially expressed genes between symbiotic states in all cell clusters were identified using DESeq2 within the FindMarkers function. Additionally, marker genes for each subcluster were identified. To highlight differences in the transcriptomes of the Immune Cell states, the top 20 genes enriched in each Immune Cell subcluster were identified and plotted (genes without annotated Pfam domains were removed).

We used the Monocle 3 package (v.1.3.7 (42)) to determine transcriptomic trajectories and outcomes for all gastrodermal/agal-hosting cells from symbiotic and aposymbiotic samples. We plotted the UMAPs of the symbiotic and aposymbiotic subsets of Gastrodermis clusters 1-5 and the Algal-Hosting gastrodermal cells and overlaid the trajectory graph, noting nodes and outcomes.

To compare expression of genes involved in lipid transport/production and nutrient cycling in gastrodermal cells from symbiotic and aposymbiotic samples, the expression of sugar transport and nitrogen cycling genes in the reclustered Gastrodermis 1 and Gastrodermis 2 cells was analyzed. First, normalized expression values of genes involved in nitrogen cycling/symbiont density control (Glutamate Dehydrogenase [*g20747*]) and lipid metabolism (Long-Chain Fatty Acid CoA Ligase [*ACSBG1*] and Acyl-Coenzyme A Thioesterase [*ACOT4.2*]) – identified in previous whole-organism RNA-seq studies (36) – were compared across all cell clusters, then across subclusters and symbiotic states in Gastrodermis 1 and 2 (functions FeaturePlot and VlnPlt in the Seurat package (37)). FeaturePlot (37) was then used to visualize the co-expression of these genes across the subclusters of Gastrodermis 1 and Gastrodermis 2 cells from the symbiotic sample (scaling the per-cell mean expression value of each gene to a maximum value of 10). To test whether the co-expression observed in the symbiotic gastrodermal cells is seen in other cell types, we also visualized co-expression of *g20747*, *ACSBG1*, and *ACOT4.2* across Epidermis 1 and Neuron 1 from symbiotic tissue (scaling per-cell mean expression to a maximum value of 10).

To identify the genes driving separation across symbiotic state within each cell cluster, the distribution of cells from a given cluster was visualized along the first 10 Principal Components (PCs) of the PCA assay that were used to define the UMAP for that cluster. An analysis of variance was used to determine whether the cells were separated by symbiotic state (symbiotic vs. aposymbiotic) along each PC. In Gastrodermis 1 and Gastrodermis 2 clusters, the top 20 genes driving the distribution of cells along each significant PC (10 genes driving each PC in the positive direction and 10 genes driving each PC in the negative direction) were selected, and genes without annotated Pfam domains were removed. In Gastrodermis 1, PCs 1-4, 6, 7, and 10 were selected for a total of 89 genes. In Gastrodermis 2, PCs 1-5, 8, 9, and 10 were selected for a total of 90 genes. Log_2_foldchange values from DESeq2 with accompanying adjusted *P* values (<0.05) for each gene were plotted in a bar graph. Genes annotated with immune-function related GO terms were noted, as were genes annotated with the Clusters of Orthologous Genes (COG) annotation ‘posttranslational modification, protein turnover, and chaperones.’

To compare *O. arbuscula* cells potentially hosting algal symbionts with algal-hosting cells from an obligate coral species (*Xenia* sp. (40)), we identified orthologous genes between *O. arbuscula* and *Xenia* implemented with the Broccoli algorithm using 14 cnidarian proteomes with default parameters (43). We re-ran Seurat normalization, PCA, and UMAP clustering on the pre-integrated *Xenia* dataset from non-regeneration samples (40) to identify 16 clusters (resolution of 0.18 and 30 dimension), including the previously characterized algal-hosting cell cluster and the other two gastrodermal cell clusters. We identified differentially expressed genes between the algal-hosting cells and the gastrodermal cells (adjusted *P* value <0.05 from the FindMarkers function, using both gastrodermal cell clusters as the comparison to the algal-hosting cell cluster), and subsetted these genes for ones that had orthologs to *O. arbuscula*. Additionally, we identified differentially expressed genes between symbiotic *O. arbuscula* cells from Gastrodermis 1/Gastrodermis 2/Gastrodermis 3/Gastrodermis 4/Algal-Hosting and aposymbiotic *O. arbuscula* cells from Gastrodermis 1/Gastrodermis 2/Gastrodermis 3/Gastrodermis 4/Algal-Hosting (adjusted *P* value <0.05 from the FindMarkers function). Gastrodermis 5 was not included because this cluster was not on the same transcriptomic trajectory in symbiotic or aposymbiotic *O. arbuscula*. We subsetted these genes to include only *Xenia* orthologs. We compared which genes among these two sets of orthologs (*Xenia*-sym-to-gastro and *Oculina-*sym-to-apo respectively) were differentially expressed using a Venn diagram (44), creating three groups of differentially expressed orthologs (DEOs). We performed Fisher Exact Tests (DEO presence/absence) (41) to identify enrichment differences across these groups of DEOs in the BP GO division.

**Supplementary References:**

1. Lajeunesse TC, Parkinson JE, Reimer JD. A genetics-based description of *Symbiodinium minutum sp. nov*. and *S. psygmophilum sp. nov*. (Dinophyceae), two dinoflagellates symbiotic with cnidaria. *J Phycol*. 2012;48(6):1380–91.

2. Enríquez S, Méndez ER, Prieto RI. Multiple scattering on coral skeletons enhances light absorption by symbiotic algae. *Limnol Oceanogr*. 2005;50(4):1025–32.

3. Scheufen T, Iglesias-Prieto R, Enríquez S. Changes in the number of symbionts and *Symbiodinium* cell pigmentation modulate differentially coral light absorption and photosynthetic performance. *Front Mar Sci*. 2017 Sep 26;4:309.

4. Vásquez-Elizondo RM, Legaria-Moreno L, Pérez-Castro MÁ, Krämer WE, Scheufen T, Iglesias-Prieto R, et al. Absorptance determinations on multicellular tissues. *Photosynth Res*. 2017 Jun 1;132(3):311–24.

5. Parada AE, Needham DM, Fuhrman JA. Every base matters: assessing small subunit rRNA primers for marine microbiomes with mock communities, time series and global field samples. *Environ Microbiol*. 2016;18(5):1403–14.

6. Apprill A, McNally S, Parsons R, Weber L. Minor revision to V4 region SSU rRNA 806R gene primer greatly increases detection of SAR11 bacterioplankton. *Aquat Microb Ecol*. 2015 Jun 4;75(2):129–37.

7. Martin M. Cutadapt removes adapter sequences from high-throughput sequencing reads. *EMBnet J*. 2011 May 2;17(1):10–2.

8. Callahan BJ, Sankaran K, Fukuyama JA, McMurdie PJ, Holmes SP. Bioconductor workflow for microbiome data analysis: From raw reads to community analyses. *F1000Res*. 2016 Nov 2;5:1492.

9. Quast C, Pruesse E, Yilmaz P, Gerken J, Schweer T, Yarza P, Peplies J, Glöcknew FO. The SILVA ribosomal RNA gene database project: improved data processing and web-based tools. *Nucleic Acids Res*. 2013 Jan 1;41(D1):D590–6.

10. Davis NM, Proctor DM, Holmes SP, Relman DA, Callahan BJ. Simple statistical identification and removal of contaminant sequences in marker-gene and metagenomics data. *Microbiome*. 2018 Dec 17;6:226.

11. Oksanen J, Simpson GL, Blanchet FG, Kindt R, Legendre P, Minchin PR, et al. vegan: Community Ecology Package. 2024.

12. Matz MV. MCMC.OTU: Bayesian analysis of multivariate counts data in DNA metabarcoding and ecology. 2016.

13. McMurdie PJ, Holmes S. phyloseq: An R package for reproducible interactive analysis and graphics of microbiome census data. *PLoS ONE*. 2013 Apr 22;8(4):e61217.

14. Bates D, Mächler M, Bolker B, Walker S. Fitting linear mixed-effects models using lme4. *J Stat Soft*. 2015 Oct 7;67(1)1-48.

15. Love MI, Huber W, Anders S. Moderated estimation of fold change and dispersion for RNA-seq data with DESeq2. *Genome Biol*. 2014 Dec 5;15(12):550.

16. Cheng H, Concepcion GT, Feng X, Zhang H, Li H. Haplotype-resolved de novo assembly using phased assembly graphs with hifiasm. *Nat Methods*. 2021 Feb;18(2):170–5.

17. Zhou C, McCarthy SA, Durbin R. YaHS: yet another Hi-C scaffolding tool. *Bioinformatics*. 2023 Jan 1;39(1):btac808.

18. Uliano-Silva M, Ferreira JGRN, Krasheninnikova K, Blaxter M, Mieszkowska N, Hall N, et al. MitoHiFi: a python pipeline for mitochondrial genome assembly from PacBio high fidelity reads. *BMC Bioinformatics*. 2023 Jul 18;24:288.

19. Pointon DL, Sims Y, Eagles W. sanger-tol/treeval [1.2.0 - Ancient Destiny-]. 2025. Available from: 10.5281/zenodo.10047653

20. Flynn JM, Hubley R, Goubert C, Rosen J, Clark AG, Feschotte C, et al. RepeatModeler2 for automated genomic discovery of transposable element families. *Proc Natl Acad Sci USA*. 2020 Apr 28;117(17):9451–7.

21. Stanke M, Bruhn W, Becker F, Hoff KJ. VARUS: sampling complementary RNA reads from the sequence read archive. *BMC Bioinformatics*. 2019 Nov 8;20:558.

22. Kim D, Paggi JM, Park C, Bennett C, Salzberg SL. Graph-based genome alignment and genotyping with HISAT2 and HISAT-genotype. *Nat Biotechnol*. 2019 Aug;37(8):907–15.

23. Gabriel L, Brůna T, Hoff KJ, Ebel M, Lomsadze A, Borodovsky M, Stanke MI. BRAKER3: Fully automated genome annotation using RNA-seq and protein evidence with GeneMark-ETP, AUGUSTUS, and TSEBRA. *Genome Res*. 2024 May 1;34(5):769–77.

24. Cantalapiedra CP, Hernández-Plaza A, Letunic I, Bork P, Huerta-Cepas J. eggNOG-mapper v2: Functional annotation, orthology assignments, and domain prediction at the metagenomic scale. *Mol Biol Evol*. 2021 Dec 1;38(12):5825–9.

25. Manni M, Berkeley MR, Seppey M, Simão FA, Zdobnov EM. BUSCO Update: Novel and streamlined workflows along with broader and deeper phylogenetic coverage for scoring of Eukaryotic, Prokaryotic, and viral genomes. *Mol Biol Evol*. 2021 Oct 1;38(10):4647–54.

26. Aguirre Carrión PJ, Williams LM, Gilmore TD. [Molecular and biochemical approaches to study the evolution of NF-κB signaling in basal metazoans.](https://www.zotero.org/google-docs/?broken=49o6yO) *Methods Mol Biol*. 2021 2366:67-91.

27. Guo H, Isserlin R, Chen X, Wang W, Phanse S, Zandstra PW, Paddison PJ, Emili A. Integrative network analysis of signaling in human CD34+ hematopoietic progenitor cells by global phosphoproteomic profiling using TiO2 enrichment combined with 2D LC-MS/MS and pathway mapping. *Proteomics*. 2013;13(8):1325–33.

28. Blum BC, Emili A. Omics Notebook: robust, reproducible and flexible automated multiomics exploratory analysis and reporting. *Bioinform Adv*. 2021 Jan 1;1(1):vbab024.

29. Ritchie ME, Phipson B, Wu D, Hu Y, Law CW, Shi W, Smyth GK. limma powers differential expression analyses for RNA-sequencing and microarray studies. *Nucleic Acids Res*. 2015 Apr 20;43(7):e47.

30. Mering C von, Huynen M, Jaeggi D, Schmidt S, Bork P, Snel B. STRING: a database of predicted functional associations between proteins. *Nucleic Acids Res*. 2003 Jan 1;31(1):258–61.

31. Shannon P, Markiel A, Ozier O, Baliga NS, Wang JT, Ramage D, Amin N, Schwikowski B, Ideker T. Cytoscape: A software environment for integrated models of biomolecular interaction networks. *Genome Res*. 2003 Nov 1;13(11):2498–504.

32. Rosental B, Kozhekbaeva Z, Fernhoff N, Tsai JM, Traylor-Knowles N. Coral cell separation and isolation by fluorescence-activated cell sorting (FACS). *BMC Cell Biol*. 2017 Aug 29;18:30.

33. Cole AG, Steger J, Hagauer J, Denner A, Ferrer Murguia P, Knabl P, Narayanaswamy S, Wick B, Montenegro JD, Technau UI. Updated single cell reference atlas for the starlet anemone *Nematostella vectensis*. *Front Zool*. 2024 Mar 18;21:8.

34. Steger J, Cole AG, Denner A, Lebedeva T, Genikhovich G, Ries A, Reїschl R, Taudes E, Lassnig M, Technau UI. Single-cell transcriptomics identifies conserved regulators of neuroglandular lineages. *Cell Rep*. 2022 Sep 20;40(12):111370.

35. Zheng GXY, Terry JM, Belgrader P, Ryvkin P, Bent ZW, Wilson R, et al. Massively parallel digital transcriptional profiling of single cells. *Nat Commun*. 2017 Jan 16;8(1):14049.

36. Rivera HE, Davies SW. Symbiosis maintenance in the facultative coral, *Oculina arbuscula*, relies on nitrogen cycling, cell cycle modulation, and immunity. *Sci Rep*. 2021 Oct 27;11:21226.

37. Hao Y, Stuart T, Kowalski MH, Choudhary S, Hoffman P, Hartman A, et al. Dictionary learning for integrative, multimodal and scalable single-cell analysis. *Nat Biotechnol*. 2024 Feb;42(2):293–304.

38. Germain PL, Lun A, Meixide CG, Macnair W, Robinson MD. Doublet identification in single-cell sequencing data using *scDblFinder* [version 2; peer review: 2 approved]. *F1000Res*. 2022;10:979.

39. Levy S, Elek A, Grau-Bové X, Menéndez-Bravo S, Iglesias M, Tanay A, et al. A stony coral cell atlas illuminates the molecular and cellular basis of coral symbiosis, calcification, and immunity. *Cell*. 2021 May 27;184(11):2973-2987.e18.

40. Hu M, Zheng X, Fan CM, Zheng Y. Lineage dynamics of the endosymbiotic cell type in the soft coral *Xenia*. *Nature*. 2020 Jun;582(7813):534–8.

41. Wright RM, Aglyamova GV, Meyer E, Matz MV. Gene expression associated with white syndromes in a reef building coral, *Acropora hyacinthus*. *BMC Genom*. 2015 May 9;16:371.

42. Trapnell C, Cacchiarelli D, Grimsby J, Pokharel P, Li S, Morse M, et al. The dynamics and regulators of cell fate decisions are revealed by pseudotemporal ordering of single cells. *Nat Biotechnol*. 2014 Apr;32(4):381–6.

43. Derelle R, Philippe H, Colbourne JK. Broccoli: Combining phylogenetic and network analyses for orthology assignment. *Mol Biol Evol*. 2020 Nov 1;37(11):3389–96.

44. Chen H. VennDiagram: Generate high-resolution Venn and Euler plots. R package v. 1.7. 3. 2022.
